# A Bayesian hierarchical model for disease mapping that accounts for scaling and heavy-tailed latent effects

**DOI:** 10.1177/09622802241293776

**Published:** 2024-12-10

**Authors:** Victoire Michal, Alexandra M Schmidt, Laís Picinini Freitas, Oswaldo Gonçalves Cruz

**Affiliations:** 1 Department of Epidemiology, Biostatistics and Occupational Health, 5620 McGill University, Montreal, Canada; 2 School of Public Health, 5622 University of Montreal, Montreal, Canada; 3 Centre de Recherche en Santé Publique, Montreal, Canada; 4 Programa de Computação Científica (PROCC), Oswaldo Cruz Foundation, Rio de Janeiro, Brazil

**Keywords:** BYM2 model, outliers, scale mixture, spatial statistics, vector-borne disease, Zika virus infection

## Abstract

In disease mapping, the relative risk of a disease is commonly estimated across different areas within a region of interest. The number of cases in an area is often assumed to follow a Poisson distribution whose mean is decomposed as the product between an offset and the logarithm of the disease’s relative risk. The log risk may be written as the sum of fixed effects and latent random effects. A modified Besag-York-Mollié (BYM2) model decomposes each latent effect into a weighted sum of independent and spatial effects. We build on the BYM2 model to allow for heavy-tailed latent effects and accommodate potentially outlying risks, after accounting for the fixed effects. We assume a scale mixture structure wherein the variance of the latent process changes across areas and allows for outlier identification. We propose two prior specifications for this scale mixture parameter. These are compared through various simulation studies and in the analysis of Zika cases from the first (2015-2016) epidemic in Rio de Janeiro city, Brazil. The simulation studies show that the proposed model always performs at least as well as an alternative available in the literature, and often better, both in terms of widely applicable information criterion, mean squared error and of outlier identification. In particular, the proposed parametrisations are more efficient, in terms of outlier detection, when outliers are neighbours. Our analysis of Zika cases finds 23 out of 160 districts of Rio as potential outliers, after accounting for the socio-development index. Our proposed model may help prioritise interventions and identify potential issues in the recording of cases.

## 1. Motivation

The first Zika cases in the Americas were identified in 2015, when it was considered a benign disease. However, in October 2015 an unprecedented increase in the number of microcephaly cases in neonates was reported in the Northeast of Brazil and was later associated with the Zika virus infection during pregnancy.^1^ The Zika virus is transmitted to humans by the bite of infected *Aedes* mosquitoes, the same vectors that transmit dengue, chikungunya and yellow fever. Dengue is the most prevalent *Aedes*-borne disease in the world and around 3.9 billion people in 129 countries are at risk of acquiring the disease.^2^ Because of climate change, the global distribution of *Aedes* mosquitoes is expanding, increasing the number of people exposed to *Aedes*-borne diseases.

In the city of Rio de Janeiro, Brazil, the first Zika epidemic occurred between 2015 and 2016, with more than 35,000 confirmed cases.^3^ The city is the second-largest in Brazil, with ∼ 6.3 million inhabitants, and its main tourist destination. Rio de Janeiro has a tropical climate and a favourable environment for the *Ae. aegypti* mosquitoes, which are highly adapted to urban settings. Despite efforts to control the vector population, the city has suffered from dengue epidemics every 3 to 4 years, in general.^4–6^ The widespread presence of the mosquito also allowed the entry and rapid dispersion of Zika and chikungunya viruses.^3^ This epidemiological scenario highlights the need for novel strategies to help design interventions that are more effective in decreasing the burden of established *Aedes*-borne diseases and preventing emerging and re-emerging arbovirus diseases from causing new outbreaks. In this sense, we propose a model that has the potential to help prioritise interventions by identifying areas with outlying risks with respect to the entire region and with respect to their neighbours, while accounting for covariates.

**Figure 1.**
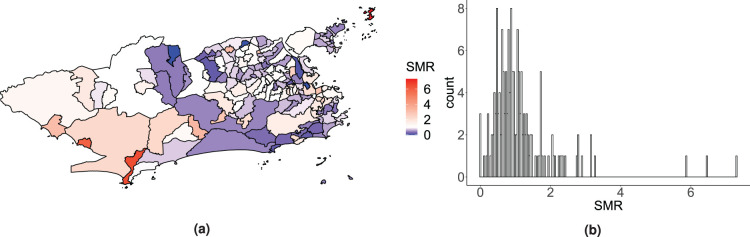
Map (a) and histogram (b) of the standardised morbidity ratio (SMR) distribution for the Zika counts across the 160 neighbourhoods of Rio de Janeiro, between 2015 and 2016.

Motivating the proposed model, we have available the Zika counts aggregated by neighbourhood for the period of the first Zika epidemic in the city of Rio de Janeiro. The data come from the Brazilian Notifiable Diseases Information System (SINAN – *Sistema de Informação de Agravos de Notificação*). In Brazil, cases attending healthcare facilities with a suspected diagnosis of Zika are reported to this system, usually by the physician. The standardised morbidity ratios (SMRs) for the Zika counts by neighbourhood during the study period are presented in Figure 1. Although the epidemic affected most of the city, some neighbourhoods seem to have been hit harder than others and some, not at all. The diversity of the territory of Rio de Janeiro is possibly an important factor influencing this. Regarding the city’s geography, for instance, there are mountains that separate different areas, which may act as a natural barrier for the spread of the disease. Additionally, Rio’s territory is heterogeneous in terms of demographic, socio-economic, and environmental characteristics that are involved in the distribution of *Aedes*-borne diseases.^7^

For this analysis, we have available the socio-development index, an index that includes indicators related to sanitation, education and income, and for which higher values represent better socio-economic conditions. In places with inadequate sanitary conditions, the female *Ae. aegypti* can more easily find any type of container filled with water to deposit her eggs. In Rio de Janeiro, a city with great social disparities, the socio-development index ranges from 0.282 (in Grumari, a neighbourhood in the West region) to 0.819 (in Lagoa, South region).^8^

### 1.1. Literature review

In the last 30 years, the field of disease mapping has experienced an enormous growth. This is because it is an important tool for decision makers to obtain reliable areal estimates of disease rates over a region of interest. Disease mapping methods, or ecological regression, further help understanding the underlying associations between covariates and the disease risk. Commonly, Bayesian hierarchical models are used to model the disease cases observed across the different areas that form a region of interest. The number of cases in an area is assumed to follow a Poisson distribution whose mean is decomposed as the product of an offset and the relative risk of the disease. Further, in the log scale, the relative risk is decomposed as the sum of covariates and latent (unobserved) areal effects. The latent components accommodate overdispersion as this decomposition of the log-relative risk can be seen as a Poisson-lognormal mixture model, if the latent effects follow a normal prior distribution.

Usually, these latent effects follow a spatial structure, a priori, such that neighbouring locations will adjust similarly after accounting for the available covariates. Indeed, it seems natural to expect that areas that are close to each other are more correlated than areas that are further apart. Let $b=[b_{1},\ldots ,b_{n}{]}^{⊤}$ be the vector of latent effects for the n areas of the region of interest. Different models have been proposed in the literature for the b ’s. First, a commonly used spatial model for the latent effects that does not accommodate outliers is the intrinsic conditional auto-regressive (ICAR) prior.^9^ Under the ICAR prior distribution, it is assumed that $b_{i}|b_{\left(-i\right)},\sigma_{b}^{2}∼N((1/d_{i})\sum_{j=1}^{n}w_{ij}b_{j},\sigma_{b}^{2}/d_{i}),\;i=1,\ldots ,n,$ where $b_{\left(-i\right)}=[b_{1},\ldots ,b_{i-1},b_{i+1},\ldots ,b_{n}{]}^{⊤}$ , $W=[w_{ij}]$ is a n×n matrix of weights, $w_{ij}$ , that defines the neighbourhood structure and where $d_{i}=\sum_{j=1}^{n}w_{ij}$ . Note that $\sigma_{b}^{2}$ is the variance parameter of the *conditional distribution* of $b_{i}$ given its neighbours. It can be shown^10^ that the joint distribution of b is proportional to $\exp\;[-(1/2\sigma_{b}^{2})b^{⊤}Qb]$ , with Q=D−W , where $D=diag(d_{i})$ . The spatial weights are often set as $w_{ij}=1$ if areas i and j share a border and $w_{ij}=0,$ otherwise. To ease the notation, let $b∼\mathrm{ICAR}(\sigma_{b},Q)$ denote the multivariate ICAR distribution. Using this common adjacency matrix, the joint ICAR distribution is not a proper multivariate normal distribution as the ‘precision’ matrix, Q , is not positive definite. One issue with the ICAR model is that it does not perform well when there is no underlying spatial structure in the data.^11^

To accommodate the presence of independent latent effects, Besag et al.^12^ proposed the so-called Besag-York-Mollié (BYM) model, where each areal latent effect is decomposed as the sum of an unstructured component and a spatially structured component. As pointed out by MacNab,^13^ this model presents an identifiability issue as the two variance components cannot be distinguished. To avoid the introduction of two random effects for each area, like in the BYM model, Leroux et al.^14^ proposed an alternative distribution for the latent spatial effects that includes a spatial dependence parameter, λ . The latter is a mixing parameter in the unit interval that allows the variance of the latent effects to be decomposed into a weighted sum between an unstructured and a spatially structured variance components. On the other hand, regarding the BYM model, Sørbye and Rue^15^ argued that scaling the spatially structured effects is essential to ease interpretation and prior assignment of the variance parameter of the latent effects, independently of the neighbourhood structure. Hence, Riebler et al.^11^ proposed the BYM2 model, that decomposes the latent effects into a weighted sum of unstructured random noises with unit variance and scaled structured components. The vector of latent spatial effects is scaled according to the neighbourhood structure. This BYM2 model is a modification of the Dean model,^16^ which is itself a modification of the BYM model. In the BYM2 model, the decomposition of the i th latent effect is as follows:

(1) $$b_{i}=\sigma_{B}\left(\sqrt{1-\lambda }\theta_{i}+\sqrt{\lambda }u_{i}^{⋆}\right),\;i=1,\ldots ,n$$

where λ∈[0,1] and θ∼N(0,I) is independent of the scaled spatially structured components, $u^{⋆}=[u_{1}^{⋆},\ldots ,u_{n}^{⋆}{]}^{⊤}∼\mathrm{ICAR}(1,Q_{⋆}).$ Let the matrix $Q_{⋆}^{-}$ be the generalised inverse of $Q_{⋆}$ , which is a scaled version of the ICAR ‘precision’ matrix, Q : $Q_{⋆}=hQ$ . The scaling factor, h , is proportional to the generalised variance that arises from an ICAR model, $h=\exp\;[(1/n)\sum_{i=1}^{n}\ln\;(Q_{ii}^{-})]$ . Note that the scaling factor only depends on the graph of the region under study. This scaled ICAR prior corresponds to $u^{⋆}=[u_{1}/\sqrt{h},\ldots ,u_{n}/\sqrt{h}{]}^{⊤},$ for u∼ICAR(1,Q). As stated by Sørbye and Rue,^15^ this scaling process allows each structured component to have a variance of approximately one. For further discussion on the scaling process, refer to Section 3.2 of Riebler et al.^11^ It results that $V(b_{i}|\sigma_{B})=\sigma_{B}^{2}[(1-\lambda )V(\theta_{i})+\lambda V(u_{i}^{⋆})]≃\sigma_{B}^{2}[(1-\lambda )\times 1+\lambda \times 1]=\sigma_{B}^{2}.$ Hence, a *marginal* variance, $\sigma_{B}^{2}$ , is defined for the latent effects and all the parameters can be interpreted for all neighbourhood structures.

Spatial heteroscedasticity is not explicitly considered in the previous models. However, it is reasonable to imagine that some areas may have abnormally high or low disease risks. Richardson et al.^17^ emphasised the importance for disease mapping models to be able to differentiate and adapt between smoothing the risk surface and capture abrupt changes in relative risks. This issue of spatial heteroscedasticity has been increasingly considered over the recent years. For instance, regarding geostatistical data, Palacios and Steel^18^ proposed a log-normal scale mixture of a Gaussian process to accommodate heavy tails.

To allow for disparities, Congdon^19^ proposes a modification of the Leroux prior by including scale mixture parameters. More specifically, Congdon^19^ assumes

(2) $$b_{i}|b_{\left(-i\right)},\kappa ,\lambda ,\sigma_{C}^{2}∼N\left(\frac{\lambda }{1-\lambda +\lambda d_{i}}\sum_{j=1}^{n}w_{ij}\kappa_{j}b_{j},\frac{\sigma_{C}^{2}}{\kappa_{i}(1-\lambda +\lambda d_{i})}\right),\;i=1,\ldots ,n$$

with $\kappa_{i}\overset{i.i.d.}{∼}\mathrm{Gamma}(\nu /2,\nu /2),\;i=1,\ldots ,n$ and $\nu ∼\mathrm{Exp}(1/\mu_{\nu }),$ for some value of $\mu_{\nu }$ fixed by the analyst. These positive parameters, $\kappa =[\kappa_{1},\ldots ,\kappa_{n}{]}^{⊤}$ , allow for discrepancies in the neighbouring estimated risks, while the usual CAR-type priors aim to locally smooth the risk surface. The scale mixture parameters are termed outlier indicators as κ<1 captures outliers. Similar to Palacios and Steel,^18^ throughout this article, the term ‘outlier’ designates any area whose variance is elevated (when $\kappa_{i}<1$ ) compared to the rest of the region. Again, for λ∈(0,1) , $\sigma_{C}^{2}$ is the variance parameter of the *conditional distribution* of $b_{i}$ given its neighbours. This implies that the interpretation of $\sigma_{C}^{2}$ differs with every spatial structure, which renders its prior assignment not straightforward and makes interpretation difficult. It can be shown^19^ that the joint distribution of the latent effects is $b|\sigma_{C}^{2},\lambda ,\kappa ∼N(0,\sigma_{C}^{2}Q_{C}^{-}),$ where the ‘precision’ matrix has diagonal elements $Q_{C_{ii}}=\kappa_{i}(1-\lambda +\lambda d_{i})$ and off-diagonal elements $Q_{C_{ij}}=-\lambda w_{ij}\kappa_{i}\kappa_{j}$ . The diagonal dominance condition^20^ states that a sufficient condition for a symmetric matrix $Q_{C}$ to be symmetric positive definite is $Q_{C_{ii}}>\sum_{j\neq i}|Q_{C_{ij}}|,\;\forall i.$ Hence, it is sufficient that λ∈[0,1) and $\lambda <\min_{i}\{1/(1-d_{i}+\sum_{j\neq i}w_{ij}\kappa_{j})\},$ for $Q_{C}$ to be a valid precision matrix. Note that if $\kappa =1_{n}$ , then Congdon’s prior is the Leroux prior, which is proper for λ∈[0,1) . This mixture differs from the commonly used normal-gamma model, as the scale mixture components appear both in the mean and in the variance of the conditional distribution. Because the scale mixture components appear in the conditional mean, areas that share a border with an outlying area give this outlier a lower weight. Let neighbouring areas i and j be outliers, and let area k be a neighbour of i and not an outlier. Then, $b_{j}$ contributes by a weight of $\kappa_{j}<1$ to the conditional mean of $b_{i}$ , whereas $b_{k}$ contributes by a factor of $\kappa_{k}>\kappa_{j}$ . This is a drawback when there are multiple outlying areas that are neighbours, as they will not borrow strength from each other.

Different from Congdon,^19^ Dean et al.^21^ addressed local discrepancies by changing the neighbouring structure according to the observed data. This approach differs from Congdon’s as it is a two-step procedure that implies changing the neighbourhood structure. Other models have been proposed to allow the strength of the spatial autocorrelation to vary over a region of interest. Corpas-Burgos and Martinez-Beneito^22^ proposed the so-called adaptive ICAR and adaptive Leroux models, which are modifications of the ICAR and Leroux models, by estimating the weights in the matrix W . The adaptive Leroux model they proposed (Corpas-Burgos and Martinez-Beneito (CB-MB) model) can be tied to Congdon’s model (2). For λ=0 , Congdon’s model yields independent latent effects with variance divided by the scaling mixture component. Similarly, when λ=0 , the CB-MB model yields independent latent effects with variance divided by the spatial weight (see e.g. Table 10 in Appendix H in the Supplemental Material). However, Corpas-Burgos and Martinez-Beneito point out that a single dataset is not enough to learn about those weights; so they suggest that their method is more suitable when modelling a multivariate outcome, where the neighbourhood structure is the same for the different outcomes. On the other hand, MacNab^23^ recently proposed a model that allows the spatial mixing parameter, λ , to change across space. This approach allows the underlying structure of the areal latent effects to differ from their neighbours, when necessary. The model proposed by MacNab differs from our proposal because it points out which structure, between the independent and spatially structured included in the BYM2 model, is more important for each region. The method proposed by MacNab does not allow for different variances across the region of interest, nor the identification of outlying areas.

The main aim of this article is to propose a method to accommodate and identify outlying areas, following a single-step inference procedure. We propose a modification of the BYM2 prior (1) that can identify outlying areas, after accounting for the effect of covariates. A scale mixture is introduced in the BYM2 model. The proposed model keeps the appealing property of parameter interpretation while capturing potentially outlying areas and allowing the neighbouring outlying areas to borrow strength from each other. Areas may be outliers with respect to the whole region of interest, namely areas with extreme disease risks; or with respect to their neighbours, termed spatial outliers. Throughout, the term ‘outlier’ refers to both types of outliers: extremes and spatial outliers. This article is organised as follows: Section 2 describes the proposed model, then a simulation study showcases the performance of the proposed model in Section 3. Additionally, the application of the proposed model to the data presented in Section 1 from the 2015-2016 Zika epidemic in the 160 neighbourhoods of Rio de Janeiro is shown in Section 3. Section 4 concludes with a discussion.

## 2. Proposed model

Let a region of interest be partitioned into n non-intersecting areas. Let $Y_{i}$ be the number of cases in area i,i=1,…,n, and $E_{i}$ , the expected number at risk in that area. The counts are modelled through the following Poisson model:

$$Y_{i}|E_{i},\mu_{i}∼P(E_{i}\mu_{i})$$

where $\mu_{i}$ denotes the relative risk in area i and $E_{i}$ is an offset. Commonly, the risk is decomposed in the log scale as follows:

$$\ln\;(\mu_{i})=\beta_{0}+x_{i}^{⊤}\beta +b_{i}$$

where $\beta_{0}$ is the overall log risk, $x_{i}$ is a p -dimensional vector with the explanatory variables in area i , associated with the p coefficients β , and $b_{i}$ is a random effect for area i . This latent effect is included in order to allow for overdispersion in the Poisson model that would otherwise assume equal mean and variance for area i . The latent areal effects can also accommodate an assumed underlying spatial structure in the data. To that end, a spatial structure is defined through the matrix $W=[w_{ij}]$ . Throughout this article, we assume that two areas are said to be neighbours if they share a border. This implies that $w_{ij}=1$ if areas i and j are neighbours and $w_{ij}=0,$ otherwise. In this setting, $d_{i}=\sum_{j=1}^{n}w_{ij}$ corresponds to the number of neighbours of area i . To model the latent areal effects accounting for such 0-1 spatial structure, we propose a modification of the BYM2 prior (1), that is, we assume

(3) $$b_{i}=\frac{\sigma }{\sqrt{\kappa_{i}}}\left(\sqrt{1-\lambda }\theta_{i}+\sqrt{\lambda }u_{i}^{⋆}\right),\;i=1,\ldots ,n$$

where σ>0 is divided by the scaling mixture component $\kappa_{i}>0$ , and where λ∈[0,1] . The component $\theta_{i}$ is assumed independent of $u_{i}^{⋆}$ . In particular, $\theta \equiv [\theta_{1},\ldots ,\theta_{n}{]}^{⊤}∼N(0,I)$ , and $u^{⋆}\equiv [u_{1}^{⋆},\ldots ,u_{n}^{⋆}{]}^{⊤}∼\mathrm{ICAR}(1,Q_{⋆})$ . Components $\theta_{i}$ and $u_{i}^{⋆}$ are termed the unstructured and the scaled structured components, respectively. Like in the BYM2 model^11^ (1), the ‘precision’ matrix is such that $Q_{⋆}=hQ$ , where the scaling factor, h , is computed from the neighbourhood structure (see Section 1.1). It results that, $V(b_{i}|\sigma ,\kappa_{i})=(\sigma^{2}/\kappa_{i})[(1-\lambda )V(\theta_{i})+\lambda V(u_{i}^{⋆})]≃(\sigma^{2}/\kappa_{i})[(1-\lambda )\times 1+\lambda \times 1]=\sigma^{2}/\kappa_{i}$ . Hence, $\sigma^{2}/\kappa_{i}$ represents the approximate *marginal* variance of the i th area’s latent effect. Moreover, the variance-covariance matrix, V , of the proposed latent effects, b , is given by $V=\sigma^{2}K^{-1}[(1-\lambda )I+\lambda Q_{⋆}^{-}]$ , where $K=diag(\kappa_{i})$ . Thus, the parameter λ represents the weight of the spatial effect in the variance of the latent process. Note that this distribution is a proper multivariate normal for small values of λ , depending on the neighbourhood structure. Indeed, the diagonal dominance condition^20^ implies that it is sufficient that λ∈[0,1) and $\lambda <\min_{i}\{1/(1-Q_{{⋆}_{ii}}^{-}+\sum_{j\neq i}|Q_{{⋆}_{ij}}^{-}|)\}$ for the covariance matrix, V , to be valid.

In a nutshell, the proposed model uses interpretable parameters to accommodate outlying areas while identifying them. The proposed model points at neighbourhoods that need heavy-tailed latent effects, through the introduction of the scale mixture components, $\kappa =[\kappa_{1},\ldots ,\kappa_{n}{]}^{⊤}$ . Area i is identified as an outlier when the posterior distribution of $\kappa_{i}$ is smaller than 1 with 95% probability. Different from Congdon’s model in equation (2), the proposed model makes use of parameters that intervene on the *marginal* distribution of the latent effects. Therefore, their prior assignment is simplified as their interpretation remains the same regardless of the neighbourhood structure. This concerns the weight of the spatial structure λ , the marginal variance $\sigma^{2}$ , as well as the scaling mixture parameters $\kappa_{1},\;\ldots ,\kappa_{n}$ when the κ ’s are assumed independent across the region.

We now compare the interpretation and roles of the scale mixture components κ in the proposed model and in Congdon’s model. To interpret the scale mixture components κ , the importance of the spatial structure in the data, measured by λ , must be taken into account. When λ=0 , both models reduce to independent latent effects without spatial structure. In that case, $\kappa_{i}<1$ only impacts the *marginal* variance of the i th latent effect and identifies an outlying area that showcases an extreme disease risk, after accounting for covariates. When λ=1 , the proposed latent effects become $b_{i}=(\sigma /\sqrt{\kappa_{i}})(u_{i}/\sqrt{h}),\;i=1,\ldots ,n$ . The κ ’s intervene on the *marginal* variances and $\kappa_{i}<1$ acts as an outlier indicator by inflating the i th *marginal* variance and hence allowing the i th effect to differ from the overall mean structure. Additionally, when λ=1 , the *conditional* distribution of the latent effects may be written as follows:

(4) $$b_{i}|b_{\left(-i\right)},\sigma^{2},\kappa ∼N\left(\frac{1}{d_{i}}\sum_{j=1}^{n}w_{ij}\sqrt{\frac{\kappa_{j}}{\kappa_{i}}}b_{j},\frac{\sigma^{2}/h}{\kappa_{i}d_{i}}\right),\;i=1,\ldots ,n$$

We compare the *conditional* distributions (2) and (4) considering the case where neighbouring areas i and j are both outliers with $\kappa_{i},\kappa_{j}<1$ and i∼j . In both distributions (2) and (4), the i th and j th *conditional* variances are inflated by $\kappa_{i}$ and $\kappa_{j}$ , respectively. Regarding the *conditional* means, in the proposed model, $\kappa_{j}/\kappa_{i}≃\kappa_{i}/\kappa_{j}≃1$ and outlying effects are allowed to borrow strength from neighbouring outliers. However, in Congdon’s model, the mutual weights of $b_{i}$ and $b_{j}$ are deflated and areas i and j contribute less to their mutual latent effects. This feature of borrowing strength in the proposed model is attractive in the case where neighbouring areas have extreme disease risks.

In the next subsection, different prior distributions are discussed for the scale mixture components.

### 2.1. Prior specification of the scale mixture component

A natural choice, and used by Congdon,^19^ is to assume

(5) $$\kappa_{i}\overset{i.i.d.}{∼}\mathrm{Gamma}\left(\nu /2,\nu /2\right),\;i=1,\ldots ,n,\;\text{and}\;\nu ∼\mathrm{Exp}(1/\mu_{\nu })$$

where the hyperparameter’s mean $\mu_{\nu }$ controls the magnitude of ν . When λ=0 , marginalising the proposed distribution (3) of the latent effect, $b_{i}$ , with respect to $\kappa_{i}$ yields a Student- t distribution with $\mu_{\nu }$ degrees of freedom, that is $t_{\mu_{\nu }}$ . The introduction of $\kappa_{i}$ hence allows for heavier tails than a Gaussian distribution for the latent effects. In this case, $\mu_{\nu }$ corresponds to choosing the degrees of freedom of the resulting t distribution, which impact the moments of the distribution as well as its tails. A large $\mu_{\nu }$ results in a distribution close to being normal, which is inadequate to capture outliers. On the other hand, $\mu_{\nu }<3$ implies a t distribution whose variance is not defined. Some simulation studies showed that setting $\mu_{\nu }=4$ performed well, which is the value suggested by Gelman et al.^24^

Another possible prior specification for the κ ’s is to borrow ideas from Palacios and Steel^18^ who proposed the inclusion of a scale mixture component in the variance of a Gaussian process. The authors suggest the usual gamma mixing is not always appropriate, as not all positive moments exist. Additionally, they point out that the t distribution that results from marginalising over the gamma scaling mixture parameters may still overestimate the overall variance and struggle to detect specific outlying areas. In particular, they assume that the scale mixture component follows a log-Gaussian process with the same spatial structure as the one defined for the main Gaussian process. Here, we propose a scaled log proper CAR prior distribution for the κ ’s. This form of discretisation of the method proposed by Palacios and Steel^18^ is applied to the latent effects, $b_{i},\;i=1,\ldots ,n$ , which include both the structured and unstructured components, in order to keep the interpretative property of the parameters. This contrasts with the method proposed by Palacios and Steel^18^ as they introduced a scale mixture only for the spatially dependent components, leaving the unstructured components untouched. Let the scale mixture components be modelled as follows:

(6) $$\begin{array}{rl} \ln\;(\kappa_{i}) & \equiv -\frac{\nu_{\kappa }}{2}+z_{i},\;i=1,\ldots ,n\\ \text{where}\;z\equiv [z_{1},\ldots ,z_{n}{]}^{⊤} & |\nu_{\kappa }∼N\left(0,\nu_{\kappa }Q_{\alpha ,⋆}^{-1}\right)\;\text{and}\;\nu_{\kappa }∼\mathrm{Exp}(1/\mu_{\nu_{\kappa }}) \end{array}$$

where $Q_{\alpha ,⋆}=hQ_{\alpha }=h_{\alpha }[D-\alpha W]$ is again a precision matrix that is scaled by $h_{\alpha }$ , which is computed based on D−αW . The parameter α guarantees $Q_{\alpha }$ to be a valid precision matrix for α∈[0,1) .^10^ For this proper distribution to be close to an ICAR prior, we impose α=0.99 . The proper CAR distribution is scaled to approximately have $V[\ln\;(\kappa_{i})|\nu_{\kappa }]≃\nu_{\kappa }\times 1$ . Similarly to Palacios and Steel,^18^ this prior implies $E(\kappa_{i}|\nu_{\kappa })≃1$ , which corresponds to a constant marginal variance across the areal latent effects, and $V(\kappa_{i}|\nu_{\kappa })=[\exp\;(V(\ln\;(\kappa_{i}|\nu_{\kappa }))-1)]\exp\;(2E(\ln\;(\kappa_{i}|\nu_{\kappa }))+V(\ln\;(\kappa_{i}|\nu_{\kappa })))≃[\exp\;(\nu_{\kappa })-1]\exp\;(-\nu_{\kappa }+\nu_{\kappa })=\exp\;(\nu_{\kappa })-1,\;\forall i$ . For $\nu_{\kappa }$ close to 0, κ is close to 1 with a small variance. A bigger $\nu_{\kappa }$ allows the κ ’s to differ greatly from 1 and to be closer to 0, when necessary. Palacios and Steel^18^ suggest that a reasonable prior mean for $\nu_{\kappa }$ is $\mu_{\nu_{\kappa }}=0.2$ . The simulation studies we conducted suggest that a sensible choice for $\mu_{\nu_{\kappa }}$ is $\mu_{\nu_{\kappa }}=0.3$ , which yields [0.2,2.4] as the 95% prior credible interval for the κ ’s. This includes $\kappa_{i}=1$ while allowing for departure from $\kappa_{i}=1$ , to accommodate a potentially outlying random effect of area i . This prior specification for the κ ’s allows the mixture components to borrow strength from neighbouring κ ’s. This may be of particular interest when outlying areas are neighbours.

### 2.2. Inference procedure

Following the specifications discussed in the previous section, the resultant posterior distributions, regardless of the prior specification for $\kappa_{i}$ , do not have a closed analytical form. Therefore, the posterior distributions are approximated through computational methods. In particular, Markov chain Monte Carlo (MCMC) methods are considered. The Hamiltonian Monte Carlo method implemented in the R package rstan^25^ is used for the simulation studies and real data application that follow. Morris et al.^26^ note that the no U-turn sampler implemented in rstan is more efficient than other MCMC samplers to obtain reliable estimates of the posterior distributions induced by the complex auto-regressive type of models that are of interest in this article.

One way to approximate a proper posterior distribution when assigning an ICAR prior is to add a sum-to-zero constraint on the parameters to distinguish them from any added constant. This is necessary due to the invariance of the ICAR distribution to the addition of a constant.^20^ The sum-to-zero constraint is applied to the spatial components of the proposed model, u , that need to be distinguished from the global intercept, $\beta_{0}$ . More precisely, we add a soft sum-to-zero constraint, that is $\sum_{i=1}^{n}u_{i}∼N(0,(n/1000{)}^{2})$ . The rstan implementation of the BYM2 model is discussed by Morris et al.^26^ and the code for the proposed model, which is a modification of the BYM2, is available in Appendix A in the Supplemental Material.

The scaling factor, h , needed in the BYM2 and in the proposed model, is computed through the R package R-INLA (integrated Laplace approximation, Rue et al.,^27^ www.r-inla.org) as explained by Riebler et al.^11^

## 3. Data analyses

In this section, we present the results of a simulation study that was conducted to assess the performance of the proposed model. The results from fitting the proposed model to data obtained from the first Zika epidemic that took place between 2015 and 2016 in Rio de Janeiro are also shown. In both cases, we consider the two parametrisations of the proposed model, which correspond to the two prior specifications of the scaling mixture components described in Section 2.1. In the simulation study and in the data application, the proposed model is compared to the Congdon’s model.^19^ Out of completeness, we also consider the two prior specifications for the κ ’s for the Congdon’s model. Namely, the Congdon’s model is fitted with the κ ’s following the original independent prior Gamma distributions (5), as well as with spatially structured κ ’s (6).

In the simulation study, we generate data for the 96 French departments and contaminate some areas. The goal is to check whether our proposed model is able to identify the generated outliers. Then, in the Zika data analysis in Rio de Janeiro, we compare the results of our proposed model to Congdon’s as well as the BYM2^11^ and Leroux^14^ models. We identify some potentially outlying districts which might be of interest to decision makers.

### 3.1. Simulation study: Neighbouring outliers in France

In this section, we present the results from a simulation study wherein some arbitrary neighbouring areas in France are contaminated into outlying areas, to assess the performance of the proposed model in comparison to the one proposed by Congdon. The design of the simulation study is inspired by Richardson et al.,^17^ where the goal is to assess the ability of the proposed model to both smooth over non-contaminated areas while capturing and identifying the contaminated ones. Richardson et al.^17^ emphasised the importance for disease mapping models to adapt to these abrupt changes in the risk surface.

**Figure 2.**
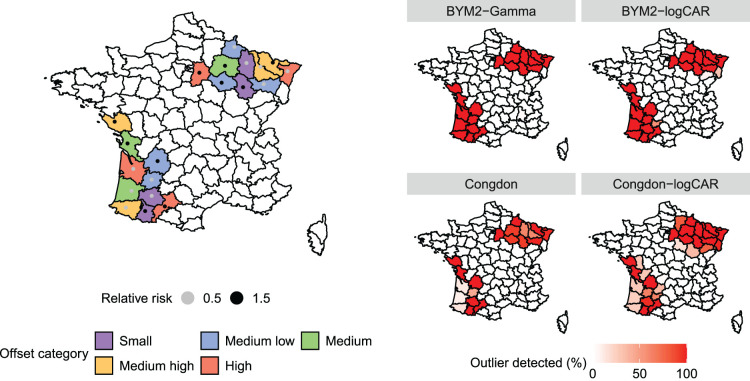
Left panel: French departments arbitrarily chosen to be outliers in the simulation study. Colours depict the offset category based on the empirical offset quantiles. The points represent the relative risk set to each outlying district. Right panel: Percentage of times, among the 100 replicates, that the outliers were identified by each model. The outliers are pointed out when $\kappa_{u}<1$ , where $\kappa_{u}$ is the upper bound of the posterior 95% credible interval of κ .

In this simulation study, 20 departments are contaminated such that two groups of 10 neighbouring outliers are created. Out of simplicity, there are no covariates included in the generating process nor when fitting the models. First, all n=96 latent effects, which correspond to log relative risks in this covariate-free simulation study, are set to 0: $b_{i}=0,\;i=1,\ldots ,n$ . Then, the offsets $[E_{1},\ldots ,E_{n}{]}^{⊤}$ are computed based on the 2019 department size estimates available on the Institut National de la Statistique et des Études Économiques (INSEE) website (https://statistiques-locales.insee.fr/#c=indicator). We define five offset categories based on the empirical offset quantiles. The first category corresponds to the smallest offsets and the fifth category, to the largest ones. The categories are termed ‘Small’ for E≤568 , ‘Medium low’ for E∈(568,906] , ‘Medium’ for E∈(906,1428] , ‘Medium high’ for E∈(1428,2399] and ‘High’ for E>2399 . Based on these categories, we select 20 departments to be outliers, such that each group of 10 neighbouring outliers contains two areas of each offset category. Within each such pair of departments, the relative risks are contaminated into outliers by setting $b_{i}=\ln\;(0.5)$ and $b_{i^{\prime }}=\ln\;(1.5)$ . The resulting outliers are mapped in the left panel of Figure 2, highlighting the offset sizes and imposed relative risks. Finally, R=100 populations of size n=96 are created according to a hierarchical Poisson model, that is, $Y_{i}∼P(E_{i}\exp\;[b_{i}])$ . As the log relative risks are fixed, the only source of randomness across the 100 replicates comes from the repeated sampling from a Poisson distribution.

Using the two-scale mixtures described in Section 2.1, the Congdon model is compared to the proposed model. The first version of the proposed model is denoted BYM2-Gamma (equation (5)) and the second, BYM2-logCAR (see equation (6)). The original Congdon model is termed Congdon, whereas the one with spatially structured scale mixture components is denoted Congdon-logCAR. For the four models, the intercept is given a quite vague prior: $\beta_{0}∼N(0,10^{2})$ and the mixing parameter, λ , is assigned a uniform, U(0,1) , prior distribution. The same $N_{+}(0,1)$ prior is considered for σ , which is a *marginal* standard deviation in the proposed model, while it is a *conditional* standard deviation in Congdon’s. Finally, in the BYM2-Gamma and Congdon models, the prior distribution for the κ ’s is described in (5) with ν∼Exp(1/4) . For the BYM2-logCAR and Congdon-logCAR parametrisations, the κ ’s follow a priori the distribution in (6) and we set ν∼Exp(1/0.3) .

The models are fitted through the R package rstan (Stan Development Team^25^). For each dataset, the MCMC procedure consists of two chains of 20,000 iterations with a 10,000 burn-in period and a thinning factor of 10. Convergence of the chains is assessed through trace plots, effective sample sizes and the $\hat{R}$ statistic (Gelman et al.^28^ and Vehtari et al.^29^).

**Figure 3.**
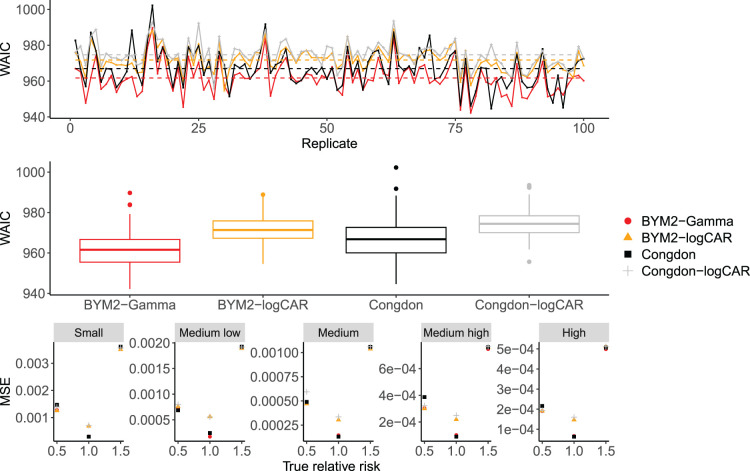
Top panel: WAIC across the 100 replicates for the proposed models and Congdon’s, in the simulation study. Dashed lines: mean WAIC for each model. Middle panel: box-plots of the WAIC values across the 100 simulation replicates for each model. Bottom panel: MSE over the 100 replicates for the proposed models and Congdon’s according to the true relative risk and the offset size. WAIC: widely applicable information criterion; MSE: mean squared error.

In terms of WAIC,^30^ for which smaller values are preferred, the proposed BYM2-Gamma model yields the smallest value among the four models, as shown in the two panels at the top of Figure 3, with an average WAIC of 962 versus 967, 972 and 975 for Congdon, BYM2-logCAR and Congdon-logCAR, respectively. In terms of MSE, Figure 3 shows that all models perform similarly: on average over the 100 replicates and all areas, the BYM2-Gamma’s MSE is 0.0003, versus 0.0004 for Congdon and 0.0005 for both models with the logCAR parametrisation.

Regarding the detection of outliers, Table 1 and the right panel of Figure 2 show how often each model accurately detects departments as outliers (sensitivity) and non-outliers (specificity), depending on the offset category. That is, the sensitivity is equal to the percentage of outliers detected among the contaminated departments over the 100 replicates. The specificity is the percentage of departments not identified as outliers among the ones whose true relative risk is equal to 1, over the 100 replicates. The definition of sensitivity and specificity are taken from Richardson et al.^17^ Area i is detected as an outlier when $\kappa_{u,i}<1$ , where $\kappa_{u,i}$ is the upper bound of the 95% posterior credible interval of $\kappa_{i}$ . The Congdon’s model with spatially structured κ ’s tends to identify more outliers than truly present in the data (overall specificity of 93%, vs. 99.9% for both BYM2-Gamma and Congdon, and 98.7 for BYM2-logCAR). More importantly, while both parametrisations of the proposed model always identify all the contaminated areas, overall, the two versions of the Congdon’s model miss 22% and 13% of the outliers. That is, the proposed spatially structured prior for the κ ’s allows the Congdon’s model to identify 10% more outliers than the model with independent mixture components.

**Table 1.** Sensitivity and specificity of the outlier detection for each model, depending on the offset size, in the simulation study.

	Offset category	BYM2-Gamma	BYM2-logCAR	Congdon	Congdon-logCAR
Sensitivity	Small	100.0	100.0	87.7	99.0
	Medium low	100.0	100.0	86.4	92.6
	Medium	100.0	100.0	66.7	75.0
	Medium high	100.0	100.0	68.0	81.2
	High	100.0	100.0	77.0	81.7
	Overall	100.0	100.0	78.1	86.8
Specificity	Small	100.0	99.2	99.9	89.2
	Medium low	99.9	96.1	99.9	90.1
	Medium	99.7	99.9	99.9	92.6
	Medium high	99.9	98.1	100.0	93.5
	High	100.0	100.0	100.0	100.0
	Overall	99.9	98.7	99.9	93.1

#### 3.1.1. Further simulation studies

To further assess the performance of the proposed model, other simulation studies were conducted. In Appendices C and D in the Supplemental Material, two simulation studies show the ability of the two versions of the proposed model to recover the true parameters when data are generated from the model itself. This suggests that the proposed model does not suffer from identifiability issues. In particular, the proposed model is able to identify and distinguish, for each district, the outlier indicators, the spatial components and the unstructured components, individually. Appendix E in the Supplemental Material presents a simulation study without contaminating any areas into outliers, which results in the proposed model performing well compared to the prior by Congdon,^19^ in terms of WAIC and in terms of outlier detection, where Congdon’s model wrongly identifies non-outlying areas as outliers. Appendix F in the Supplemental Material presents the results from a simulation study where arbitrary distant areas in France are contaminated into outliers. Again, the goal is to assess the ability of the proposed model to identify these outliers. As discussed in Section 2, in that scenario where outliers are far from each other, the proposed model performs similarly to the Congdon’s model. To show that the performance of the proposed model is independent of the neighbourhood structure under study, we present in Appendix G in the Supplemental Material the results from two simulation studies that use the map of Rio de Janeiro, where some districts are contaminated into outliers. A third simulation study shown in Appendix G.3 in the Supplemental Material aims to resemble the data analysis presented in Section 3.2, wherein a covariate is included, and relative risks vary more over the region of interest. We found that the proposed model performed better in identifying the outliers, compared to the Congdon’s model.

### 3.2. Cases of Zika during the 2015-2016 epidemic in Rio de Janeiro

The total numbers of cases of Zika were recorded across the 160 neighbourhoods of Rio de Janeiro during the first epidemic, which took place between 2015 and 2016. Let $Y_{i}$ be the disease count in district i=1,…,160. A hierarchical Poisson model is fitted to these data with offsets, E , computed from, P , the areal population sizes, $E_{i}=P_{i}(\sum_{j}Y_{j}/\sum_{j}P_{j})$ . We consider a socio-development index, x , as an explanatory variable for the number of cases. Identifying districts with potentially outlying risks, after accounting for the covariate, may be useful for decision makers to understand how to prevent Zika and where to start from. The distribution of Zika is described through a map and a histogram of the SMR, Y/E , in Figure 1 in Section 1. Some districts seem to present different SMR values than the mean surface, such as the island Paquetá, Barra de Guaratiba and Pedra de Guaratiba, with SMRs of 7.3, 6.5 and 5.9, respectively. In the lower tail of the SMR distribution, three districts did not record any cases and thus present null SMRs, namely Gericinó, Vasco da Gama and Parque Colúmbia. However, the SMR being an exploratory tool, one cannot conclude that high or low SMR values necessarily indicate outlying districts. Therefore, we are interested in comparing which districts are identified as potential outliers, after accounting for the socio-development index, by the two versions of the proposed model and Congdon’s. The same priors are defined for the parameters as in the simulation study presented in Section 3.1 and the two versions of the proposed model and Congdon’s are again denoted BYM2-Gamma, BYM2-logCAR, Congdon and Congdon-logCAR. We further compare the performance of the four models to the BYM2 and Leroux models which do not accommodate potential outliers.

All models are fitted in rstan (Stan Development Team, 2020) with two chains of 20,000 iterations thinned by 10 and of which 10,000 are burnt. As assessed by the trace plots, the effective sample sizes and the $\hat{R}$ statistics, the two chains have mixed well for all six models and convergence is attained. Appendix B in the Supplemental Material presents the trace plots, effective sample sizes and $\hat{R}$ statistics for a selection of parameters from the two parametrisations of the proposed model. The proposed BYM2-Gamma model took 15 min to run while the proposed BYM2-logCAR needed 11 min. In comparison, Congdon’s model converged in 22 min and the Congdon-logCAR, in 11 min.

**Figure 4.**
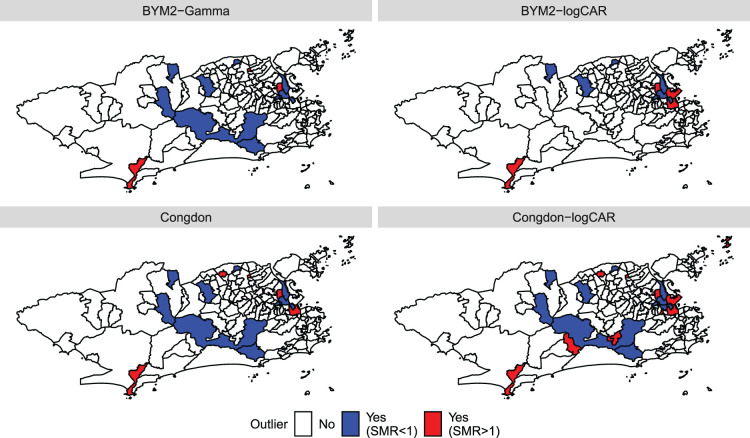
Maps of the outliers indicated by each model when analysing the Zika counts. The outliers are pointed out when $\kappa_{u}<1$ , where $\kappa_{u}$ is the upper bound of the posterior 95% credible interval of κ . The outliers on the lower tail are distinguished from the ones on the upper tail of the standardised morbidity ratios (SMR) distribution.

The results from the fitted models are presented in Table 2 and Figure 4. In terms of WAIC, the proposed BYM2-Gamma model performs best among the six considered. There is an important performance gain when accommodating outliers (BYM2-Gamma, BYM2-logCAR, Congdon and Congdon-logCAR: 1335, 1342, 1337 and 1339, respectively, vs. BYM2 and Leroux: 1371 and 1374, respectively). Congdon’s prior does not seem to perform significantly worse than the BYM2-Gamma model. Interestingly, even though the proposed model has 160 more parameters than Congdon’s, its effective number of parameters is similar (80 vs. 81). The models are further compared in terms of MSE, where $\mathrm{MSE}=(1/N)\sum_{i=1}^{N}(Y_{i}-{\hat{Y}}_{i}{)}^{2},$ where ${\hat{Y}}_{i}$ is the fitted value, that is, the estimated mean of the posterior predictive distribution. All models yield similar values, between 243.5, for the Congdon-logCAR model, and 245.7 for the Leroux model.

**Table 2.** Results from the analysis of Zika reported cases in Rio de Janeiro in 2015-2016. Model assessment (WAIC) and parameter posterior summaries: posterior mean and 95% credible interval (CI) for BYM2, BYM2-logCAR, BYM2-Gamma, Congdon and Leroux.

	BYM2	BYM2-logCAR	BYM2-Gamma	Congdon	Congdon-logCAR	Leroux
Model fit						
WAIC	1371.2	1342.3	1335.6	1337.5	1339.2	1373.9
$p_{W}$	88.6	82.3	80.0	81.0	81.1	89.2
MSE	244.8	243.7	244.1	244.3	243.5	245.7
Parameters’ posterior summaries						
	Mean (95% CI)	Mean (95% CI)	Mean (95% CI)	Mean (95% CI)	Mean (95% CI)	Mean (95% CI)
$\beta_{0}$	1.6 (0.4, 2.8)	2.5 (1.3, 3.5)	2.5 (1.7, 3.4)	2.4 (1.4, 3.2)	2.0 (1.0, 3.0)	1.2 (−0.1, 2.4)
β	−2.8 (−4.8, −0.8)	−4.2 (−5.8, −2.3)	−4.3 (−5.6, −2.9)	−4.0 (−5.4, −2.6)	−3.7 (−5.1, −1.9)	−1.9 (−4.1, −0.1)
λ	0.7 (0.4, 0.9)	0.6 (0.2, 0.9)	0.7 (0.3, 0.9)	0.8 (0.5, 0.9)	0.6 (0.2, 0.9)	0.6 (0.2, 0.9)
σ	0.8 (0.7, 0.9)	0.4 (0.3, 0.5)	0.4 (0.3, 0.5)	0.6 (0.4, 0.8)	0.6 (0.4, 0.8)	1.2 (0.9, 1.5)
ν	–	1.4 (0.7, 2.3)	2.2 (1.4, 3.3)	1.9 (1.3, 2.8)	1.7 (0.9, 2.9)	–

Regarding the intercept, $\beta_{0}$ , the proposed models and Congdon’s give similar results, whereas the Leroux and BYM2 models yield smaller posterior means and lower credible interval bounds. This is probably due to the difference in the spatial effects that are allowed to be more extreme in the Congdon, Congdon-logCAR, BYM2-Gamma and BYM2-logCAR models. All six models indicate a negative relationship between the development index and the risk of Zika, with negative posterior 95% credible intervals for β that do not include 0. We cannot directly compare the parameters λ and σ between the BYM2-type models and Leroux-type priors, as these lie in the *marginal* and *conditional* distributions of the latent effects, respectively. Marginally, the BYM2-type models yield similar weights of the spatially structured components on the latent effects (posterior means for λ of 0.6 and 0.7). For the Leroux-type models, the point estimates for λ show slightly more difference (e.g. 0.6 for Leroux and 0.8 for Congdon). This difference may be due to the presence of outliers in the data, which results in the Leroux model finding more random noise in the latent effects. The same observation can be made for the marginal and conditional standard deviation, σ , regarding the BYM2-type models and the Leroux-type models, respectively. The posterior credible interval for σ is significantly higher in the BYM2 model compared to the two parametrisations of the proposed model, and in the Leroux model compared to the two versions of Congdon’s model. Indeed, the proposed models are able to estimate a smaller overall variance for the latent effects, which is then adjusted through the κ ’s when needed. Finally, it can be noted that there seems to be enough information in the data to learn about the hyperparameter ν . This parameter was assigned a prior mean of 4 and prior 95% credible interval of [0.1,14.7] for the BYM2-Gamma and Congdon models and resulted in posterior means of about 2 and posterior 95% credible intervals of about [1,3] . The BYM2-logCAR and Congdon-logCAR models assigned an exponential distribution with mean 0.3 for ν , inducing a prior 95% credible interval of [0.0,1.1] , and yielded posterior credible intervals of [0.7,2.3] and [0.9,2.9] , showing the need for some κ ’s to be different from 1, a posteriori.

We now focus on the outliers detected by the proposed models and Congdon’s, as shown in Figure 4. District i is again found to be a potential outlier, after accounting for the socio-development index, if $\kappa_{u,i}$ , the upper bound of the posterior 95% credible interval of $\kappa_{i}$ , is below 1. In Figure 4, the blue and red coloured districts help distinguish the detected outliers on the lower tail of the SMR distribution from the ones on the upper tail. After accounting for the socio-development index, some districts are pointed out by the four models, such as Gericinó, Parque Colúmbia, Vasco da Gama and Maré, on the lower tail of the SMR distribution, Barra de Guaratiba and Bonsucesso, on the upper tail. However, Congdon’s model and both versions of the proposed approach do not point out Paquetá in the upper tail, whereas the Congdon-logCAR model detects it. This may be explained by the offset size of Paquetá, which is among the smallest in the entire region of Rio. Note, however, that the BYM2-Gamma model is close to identifying Paquetá as an outlier as it results in $\kappa_{u}=1.04$ for this district. Neither of the four models identify Pedra de Guaratiba, which has a high SMR, as shown in Figure 1. Interestingly, the district of São Cristóvão is detected as an outlier by all models except the BYM2-Gamma model, with $\kappa_{u}=1.2$ . The BYM2-logCAR and Congdon’s models detect few more potential outliers than the BYM2-Gamma model. Our simulations have shown that the BYM2-logCAR and Congdon models tend to detect non-outliers more often than the BYM2-Gamma model. We believe that this explains the differences in the outliers identified after accounting for the socio-development index.

## 4. Discussion

In this article, we propose a disease mapping model that is able to identify areas with potentially outlying disease risks, after accounting for the effects of covariates. Outliers refer to areas with extreme risks – on the tail of the risk distribution – as well as spatial outliers, after accounting for covariates. Spatial outliers correspond to areas whose risk differs from their neighbours, after accounting for covariates. The proposed model is a scale mixture of the BYM2 model.^11^ Two different prior specifications are proposed for the scale mixture components in order to compare independent components and spatially structured components. Our model allows for a straightforward interpretation of the parameters, that is common to every data application, while accommodating outliers. The parameters’ interpretation is eased by the scaling process of the latent spatially structured components.^15^

A simulation study presents the performance of the two versions of the proposed model compared to the one by Congdon,^19^ as well as a version of Congdon’s model that uses our proposed spatially structured mixture components. The neighbourhood structure of France is used and the latent effects of some neighbouring departments are contaminated to control the presence of outliers. The BYM2-Gamma version of the proposed model always performs best in terms of WAIC and in terms of MSE. Regarding the detection of outliers, the two versions of the proposed model always identify the contaminated departments, compared to the two parametrisations of Congdon’s model that miss up to 33% of the outliers. Additionally, the BYM2-Gamma version of the proposed model does not detect non-contaminated districts. Finally, in all of our simulation studies, the proposed model always performs at least as well as Congdon’s, and often better, both in terms of WAIC, MSE and of outlier identification (see e.g. Appendices F and G in the Supplemental Material).

The cases of Zika that were recorded in Rio de Janeiro during the first 2015-2016 epidemic are analysed using the two parametrisations of the proposed model as well as the model by Congdon^19^ and its version with spatially structured mixture components, the BYM2^11^ and the Leroux prior.^14^ All six models find that there is a fairly strong negative association between the socio-development index and the number of cases, meaning that richer districts have lower disease risks. This finding is consistent with previous studies conducted in Rio de Janeiro, one investigating the first chikungunya epidemic in the city^7^ and another also investigating Zika, but using a different methodological approach.^31^ These studies, including ours, indicate that improving sanitary conditions and reducing socio-economic disparities are of paramount importance to fight *Aedes*-borne diseases.

**Figure 5.**
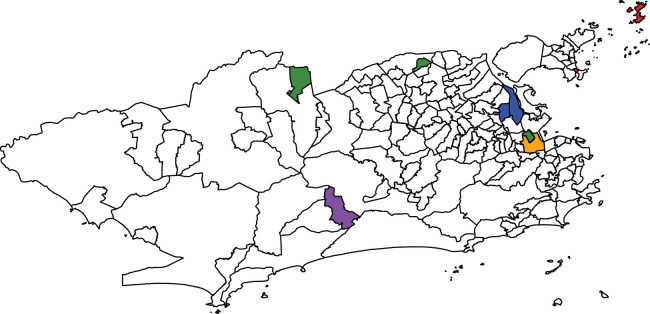
Map highlighting some districts identified as outliers by at least one model when analysing the Zika counts. Orange: São Cristóvão; red: districts with small offsets; blue: districts whose population sizes increased significantly after the 2010 census; purple: districts combining both characteristics; and green: districts with zero cases recorded.

After accounting for the effect of the socio-development index, some neighbourhoods are detected as potential outliers by the proposed models and Congdon’s, both in the lower and upper tails of the number of cases’ distribution across the districts. Out of the 23 neighbourhoods identified as outliers, irrespective of the model, the proposed models BYM2-logCAR and BYM2-Gamma identified 11 (47.8%) and 14 (60.9%), respectively. The four models do not always point out the same districts as potential outliers. One possible explanation for that is the small offset sizes of some districts. The simulation study with neighbouring outliers showed that, when the offset is small, the models that impose a spatially structured prior on the scaling mixture components tend to accurately identify outlying areas more often than the models with a priori independent mixture components. Regarding the analysis of Zika cases, Figure 5 shows in red and purple the districts identified as outliers by at least one of the four models and whose offsets are among the smaller 5%. For example, based on the results from the second simulation study, it is possible that, when analysing the Zika counts, Camorim (purple) and the island Paquetá (red) are missed by the BYM2-Gamma and Congdon models while they are pointed out by the Congdon-logCAR model (Figure 4) because of their smaller offset sizes (Figure 5).

Figure 5 highlights in green the districts with zero Zika cases recorded between 2015 and 2016: Parque Colúmbia, Gericinó and Vasco da Gama. These three districts are pointed out as outliers by the four models, as shown in Figure 4. One potential explanation for these zero recorded cases is that when the disease appeared for the first time in 2015, it was not immediately identified as Zika. Further, there is evidence that epidemics in Rio de Janeiro tend to spread starting from the north-east of the city.^3^ It is then possible that when the authorities began registering the Zika cases, there were no cases to record in the two northern districts highlighted in blue, Parque Colúmbia and Gericinó. Another potential reason is that it is not uncommon in Rio de Janeiro for a person to report as their neighbourhood of residence a neighbourhood that actually shares a border with the one where they actually live. For instance, Parque Colúmbia and Gericinó are relatively new districts and the population might not yet be used to naming them as their districts of residence. Similarly, a person living in Vasco da Gama (southern green district) may report São Cristóvão (orange) as their district. This would artificially cause Vasco da Gama to record zero cases and be detected as a potential outlier. Further, if a given district is accounting for a proportion of the cases that are in fact from the neighbouring areas (e.g. São Cristóvão), this would artificially increase the risk of this district. In fact, Figure 4 shows that São Cristóvão is pointed out as a potential outlier by all models but the BYM2-Gamma. Therefore, the inaccurate information on the district of residency may artificially create outliers.

Finally, artificial outliers may be caused by inaccurate information on the areal population sizes used to compute the offsets. While the disease counts were recorded during 2015-2016, the population sizes were extracted from the previous census, dating from 2010. Between 2010 and 2015-2016, the population sizes may have increased in some districts, without being reflected in the offsets in this analysis, causing the artificial detection of increased disease risks. Figure 5 highlights in blue and purple the districts identified as potential outliers and whose sizes have largely increased since 2010, according to more recent aerophotogrammetry flights by the Health Secretariat of the city. The eastern blue districts are pointed out as outliers by all four models in Figure 4. Further investigating these districts would help determine whether they do present outlying disease risks or if they are artificial outliers. An interesting side effect of the proposed model seems to be that by identifying outliers and further investigating the results, the authorities might better understand the population dynamics in the region of interest, in between censuses, and identifying potential issues in the accurate recording of cases.

We suggest exploring both prior specifications for the scaling mixture components, using the proposed and Congdon’s models, and further investigation on the detected districts should be conducted by decision makers and experts to fully comprehend the detected outlying behaviours. Additionally, besides looking at the posterior summary of $\kappa_{i}$ the estimated relative risks should also be studied. This is because $\kappa_{i}$ might be estimated smaller than 1 with high probability, but the posterior summary of the relative risk might include 1. This is the case for the district Benfica, when analysing the Zika counts. Benfica is found to be a potential outlier only by the Congdon-logCAR model, while all models estimate its relative risk to be close to 1 (see Table 2 in Appendix B in the Supplemental Material). Therefore, we would not conclude that Benfica is an outlier with respect to the number of Zika cases recorded during the first epidemic.

Also, it is important to emphasise that some socio-environmental factors that influence the burden and distribution of *Aedes*-borne diseases may be heterogeneous within the districts, our spatial unit of analysis. For example, the same district may have areas with *favelas* (slums) and areas with middle and upper class condominiums. The socio-development index will not capture this intra-district social inequality, and a recent study showed evidence about the presence of socio-economic inequalities in the distribution of dengue, Zika and chikungunya in two Latin American cities.^32^ Another possibility is the presence of large potential breeding sites, such as dumps and vacant lots. It is also worth mentioning that spatial confounding, which is beyond the scope of this work, is a potential issue that may affect the estimated latent effects^33,34^ and identified potential outliers. Hence, interpretation of the results should be done with care.

To conclude, we believe our proposed model to be useful to decision makers. First, the parameters’ interpretation eases the use of our model regardless of the data spatial structure. This may help decision makers to create a systematic procedure to analyse data with our proposed model, in which non-informative priors for the parameters could be defined for any spatial structure. Then, the introduction of scaling mixture components improves the recovering of the observed and potentially outlying disease risks, as assessed by the model performance criteria (WAIC and MSE). Finally, these mixture components together with high estimated risk ratios help identify all the potential outlying areas in which interventions may need to be prioritised.

## Supplemental Material

sj-pdf-1-smm-10.1177_09622802241293776 - Supplemental material for A Bayesian hierarchical model for disease mapping that accounts for scaling and heavy-tailed latent effects Supplemental material, sj-pdf-1-smm-10.1177_09622802241293776 for A Bayesian hierarchical model for disease mapping that accounts for scaling and heavy-tailed latent effects by Victoire Michal, Alexandra M Schmidt, Laís Picinini Freitas and Oswaldo Gonçalves Cruz in Statistical Methods in Medical Research

## Data Availability

The Zika and the population data analysed in this study come from the Brazilian Notifiable Diseases Information System (SINAN – Sistema de Informação de Agravos de Notificação) and the Brazilian Institute of Geography and Statistics (IBGE – Instituto Brasileiro de Geografia e Estatística), respectively, and are publicly available at the Rio de Janeiro Secretariat of Health website (http://www.rio.rj.gov.br/dlstatic/10112/7079759/4197436/ZIKASE2015.pdf and http://www.rio.rj.gov.br/dlstatic/10112/10617973/4260330/ZIKASE2016.pdf, for 2015 and 2016, respectively). Note that SINAN reflects data from the public health system (SUS – Sistema Único de Saúde) only, which does not include data from private hospitals and health plans. The sociodevelopment index data come from the Instituto Pereira Passos and can be found at www.data.rio.
