# Supplementary material for: A Bayesian hierarchical model for disease mapping that accounts for scaling and heavy-tailed latent effects
Source: Stat Methods Med Res. 2024 Dec 10;34(2):307–21. doi: 10.1177/09622802241293776 (PMC11874469; doi:10.1177/09622802241293776)
Supplement: sj-pdf-1-smm-10.1177_09622802241293776 - Supplemental material for A Bayesian hierarchical model for disease mapping that accounts for scaling and heavy-tailed latent effects [file sj-pdf-1-smm-10.1177_09622802241293776.pdf]

Supplementary material for *"A Bayesian hierarchical model for disease mapping that accounts for scaling and heavy-tailed latent effects"*

August 16, 2024

A Stan code for the proposed model

The stan code used to fit the proposed BYM2-Gamma model in the simulation studies (section 3.1, Appendices C, D, E, F and G) and in the analysis of the Zika epidemic in Rio de Janeiro (section 3.2) is presented below.

Listing 1: Stan code for the BYM2-Gamma proposed model

```
data {
  int<lower=1> N; // Number or areas
  int<lower=1> N_edges; // Total number of neighbours in the region
  int<lower=1> p; // General case where there are p covariates, excluding the intercept
  matrix[N,p] X;
  int<lower=1, upper=N> node1[N_edges]; // vectors of neighbourhood
  int<lower=1, upper=N> node2[N_edges]; // structure
  int<lower=0> y[N]; // Zika counts
  vector<lower=0>[N] log_E; // offset
  real<lower=0> scaling_factor; // to scale the variance of the latent effects
}

parameters {
  real beta0; // intercept
  vector[p] beta; // Fixed effects
  real<lower=0> sigma; // marginal standard deviation
  real<lower=0, upper=1> lambda; // mixing parameter
```

```
  vector[N] theta; // unstructured components
  vector[N] s; // spatially structured components
  vector<lower=0>[N] kappa; // outlier indicator
  real<lower=0> nu; // parameter included in the prior for each kappa_i
}

transformed parameters {
  vector[N] convolved_re; // complete latent effect
  for(i in 1:N){ convolved_re[i] = sqrt(1 - lambda) * theta[i] + sqrt(lambda/scaling_factor) * s[i]; }
}

model {
  for(i in 1:N)
   y[i] ~ poisson_log(log_E[i] + beta0 + X[i,]*beta + convolved_re[i] * (sigma/sqrt(kappa[i])) );

  target += -0.5 * dot_self(s[node1] - s[node2]); // Prior for the spatially structured components
  sum(s) ~ normal(0, 0.001 * N); // Soft sum-to-zero constraint to be able to have an intercept

  for(j in 1:p){ beta[j] ~ normal(0.0, 10.0); }

  beta0 ~ normal(0.0, 10.0);
  theta ~ normal(0.0, 1.0);
  sigma ~ normal(0.0, 1.0);
  lambda ~ uniform(0.0, 1.0);
  kappa ~ gamma(nu/2.0,nu/2.0);
  nu ~ exponential(1.0/4.0);
}

generated quantities {
  vector[N] mu_log;
  vector[N] lik;
  for(i in 1:N){
    mu_log[i]=log_E[i] + beta0 + X[i,]*beta + sigma*convolved_re[i]/sqrt(kappa[i]);
    lik[i] = exp(poisson_log_lpmf(y[i] | mu_log[i])); // likelihood to compute the WAIC
  }
}
```

B Additional results for the analysis of Zika cases recorded in Rio de Janeiro during the 2015-2016 epidemic

In this section, we present additional results regarding the analysis of Zika cases summarised in Section 3.2. First, we display the trace plots, effective sample sizes and $\widehat{R}$ statistics for a few selected parameters of the two parametrisations of the proposed model, when fitted to the data application in Section 3.2. For the mixture components, κ's, we select the ones that produced the best and the worst convergence diagnostics. Then, Tables 2 and 3 present the posterior summaries for the κ's and relative risks of all districts found to be potential outliers according to at least one model.

	BYM-Gamma		BYM2-logCAR	
	ESS	$\widehat{R}$	ESS	$\widehat{R}$
κ_{92}	1305	1.000	1614	0.999
κ_{13}	2000	0.999	2838	0.999
λ	1958	1.009	1211	1.007
ν	2000	1.000	1817	1.000
σ	1912	1.001	1987	1.004

Table 1: Effective sample sizes (ESS) and $\widehat{R}$ statistics for some parameters when fitting the two parametrisations of the proposed model to the Zika data. κ_{13} and κ_{92} were chosen because they produced the best and the worst convergence diagnostics.

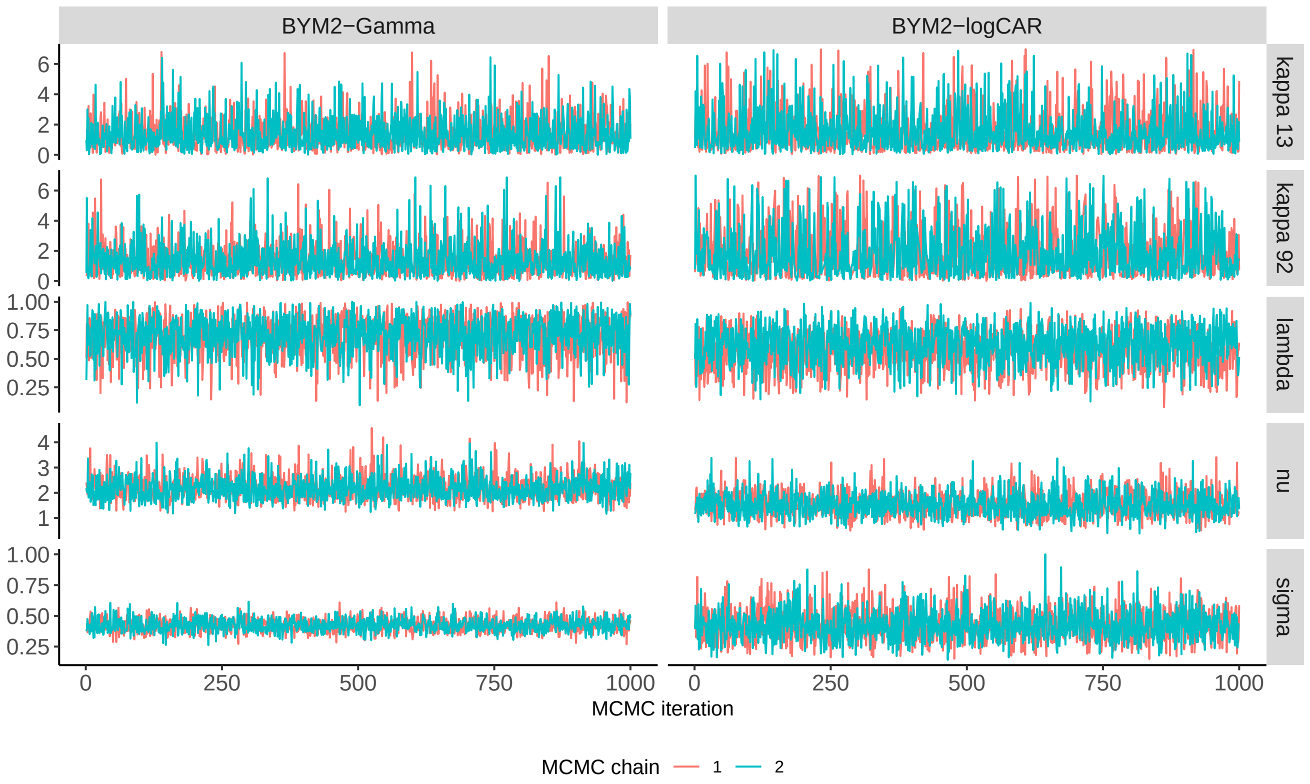

Figure 1: Trace plots for some parameters when fitting the two parametrisations of the proposed model to the Zika data. κ_{13} and κ_{92} were chosen because they produced the best and the worst convergence diagnostics.

	κ: mean (95% CI)				RR: mean (95% CI)			
#	BG	BLC	C	CLC	BG	BLC	C	CLC
10	0.38 (0.03,1.19)	**0.27 (0.03,0.95)**	**0.27 (0.01,0.97)**	**0.21 (0.04,0.61)**	2.07 (1.85,2.3)	2.08 (1.87,2.32)	2.07 (1.85,2.29)	2.08 (1.85,2.31)
113	0.3 (0.02,1.02)	0.38 (0.04,1.43)	**0.17 (0.01,0.58)**	**0.25 (0.04,0.72)**	3.12 (2.87,3.39)	3.12 (2.88,3.38)	3.13 (2.87,3.4)	3.12 (2.87,3.38)
115	**0.18 (0.01,0.69)**	0.43 (0.07,1.24)	**0.06 (0,0.2)**	**0.23 (0.07,0.53)**	0.47 (0.43,0.52)	0.48 (0.43,0.52)	0.47 (0.43,0.52)	0.48 (0.44,0.52)
116	0.52 (0.05,1.66)	0.48 (0.04,1.87)	0.51 (0.02,2.16)	**0.28 (0.03,0.99)**	2.03 (1.82,2.26)	2.04 (1.82,2.28)	2.03 (1.81,2.26)	2.04 (1.83,2.27)
117	0.59 (0.04,1.85)	0.53 (0.05,2.05)	0.46 (0.02,2.15)	**0.28 (0.03,0.94)**	2.3 (2.02,2.59)	2.3 (2.04,2.59)	2.3 (2.02,2.59)	2.31 (2.04,2.6)
12	1.12 (0.08,3.5)	0.3 (0.03,1.1)	1.02 (0.05,3.84)	**0.23 (0.03,0.76)**	0.88 (0.74,1.03)	0.86 (0.72,1.01)	0.89 (0.75,1.04)	0.87 (0.73,1.02)
127	**0.21 (0.01,0.66)**	0.3 (0.03,1.02)	**0.18 (0.01,0.72)**	**0.26 (0.04,0.84)**	0.37 (0.29,0.45)	0.37 (0.29,0.45)	0.37 (0.29,0.45)	0.37 (0.29,0.45)
129	0.48 (0.03,1.59)	0.48 (0.04,1.74)	0.32 (0.02,1.15)	**0.28 (0.04,0.89)**	3.04 (2.15,4.12)	3.08 (2.17,4.17)	3.05 (2.14,4.15)	3.06 (2.11,4.14)

13	0.34 (0.03,1.04)	0.29 (0.01,1.22)	0.36 (0.02,1.16)	**0.23 (0.01,0.84)**	7.25 (6.18,8.48)	7.27 (6.2,8.51)	7.22 (6.15,8.37)	7.23 (6.11,8.43)
135	**0.17 (0.01,0.58)**	**0.3 (0.03,0.95)**	**0.09 (0,0.37)**	**0.21 (0.04,0.66)**	0.23 (0.14,0.35)	0.25 (0.15,0.37)	0.23 (0.14,0.35)	0.25 (0.16,0.37)
142	**0.29 (0.02,0.91)**	0.38 (0.04,1.24)	**0.21 (0.01,0.74)**	**0.27 (0.04,0.81)**	0.44 (0.39,0.5)	0.44 (0.39,0.5)	0.44 (0.39,0.49)	0.44 (0.39,0.5)
152	**0.2 (0.01,0.64)**	**0.26 (0.02,0.99)**	**0.17 (0.01,0.57)**	**0.17 (0.02,0.55)**	6.35 (5.35,7.4)	6.35 (5.34,7.42)	6.36 (5.37,7.39)	6.36 (5.33,7.42)
155	**0.11 (0.01,0.35)**	**0.2 (0.03,0.66)**	**0.07 (0,0.26)**	**0.15 (0.03,0.44)**	0.23 (0.17,0.29)	0.23 (0.18,0.3)	0.23 (0.17,0.3)	0.23 (0.18,0.3)
157	**0.05 (0,0.16)**	**0.15 (0.02,0.48)**	**0.03 (0,0.09)**	**0.08 (0.02,0.22)**	0.11 (0.09,0.13)	0.11 (0.09,0.14)	0.11 (0.09,0.13)	0.11 (0.09,0.14)
158	**0.02 (0,0.08)**	**0.09 (0.01,0.34)**	**0.01 (0,0.05)**	**0.06 (0.01,0.19)**	0.01 (0,0.06)	0.04 (0,0.11)	0.01 (0,0.05)	0.03 (0,0.1)
159	**0.01 (0,0.06)**	**0.04 (0,0.16)**	**0.01 (0,0.05)**	**0.03 (0,0.1)**	0.01 (0,0.03)	0.01 (0,0.04)	0 (0,0.03)	0.01 (0,0.04)
160	**0.05 (0,0.21)**	**0.04 (0,0.2)**	**0.08 (0,0.78)**	**0.03 (0,0.14)**	0 (0,0.03)	0 (0,0.02)	0.01 (0,0.04)	0 (0,0.03)
39	**0.27 (0.01,0.88)**	**0.24 (0.03,0.79)**	**0.21 (0.01,0.82)**	**0.19 (0.03,0.6)**	0.48 (0.39,0.58)	0.47 (0.39,0.57)	0.48 (0.4,0.58)	0.48 (0.39,0.57)
4	0.85 (0.06,2.59)	**0.25 (0.03,0.89)**	0.7 (0.04,2.69)	**0.18 (0.03,0.55)**	1.7 (1.48,1.94)	1.72 (1.5,1.96)	1.69 (1.47,1.93)	1.72 (1.5,1.95)
40	**0.26 (0.01,0.9)**	**0.26 (0.03,0.9)**	**0.17 (0.01,0.58)**	**0.17 (0.02,0.52)**	2.4 (2.11,2.7)	2.4 (2.12,2.69)	2.4 (2.14,2.67)	2.4 (2.14,2.69)
57	0.57 (0.05,1.78)	0.48 (0.04,1.75)	0.31 (0.01,1.12)	**0.32 (0.04,0.98)**	1.76 (1.43,2.13)	1.78 (1.44,2.15)	1.76 (1.39,2.14)	1.77 (1.44,2.15)
75	**0.21 (0.01,0.69)**	0.29 (0.03,1.03)	**0.16 (0.01,0.55)**	**0.21 (0.03,0.68)**	2.69 (2.26,3.16)	2.7 (2.27,3.16)	2.7 (2.29,3.16)	2.7 (2.28,3.16)
92	**0.25 (0.01,0.91)**	0.35 (0.03,1.28)	**0.17 (0.01,0.59)**	**0.31 (0.03,0.99)**	2.65 (1.83,3.59)	2.59 (1.77,3.59)	2.67 (1.81,3.67)	2.64 (1.82,3.61)

Table 2: Posterior summaries (mean and 95% credible interval) for κ and RR, the relative risk, estimated in the Zika data analysis summarised in Section 3.2 of the paper. The results are shown only for the 23 districts out of 160 that are found to be potential outliers, according to both versions of the proposed model and Congdon's. #: District number (see Table 3 below); BG: BYM2-Gamma; BLC: BYM2-logCAR; C: Congdon; CLC: Congdon-logCAR; Boldface: summaries for κ that do not include 1.

#	District name	#	District name	#	District name
10	São Cristóvão	13	Paquetá	160	Gericinó
113	Costa Barros	135	Vila Militar	39	Manguinhos
115	Jacarepaguá	142	Senador Camará	4	Caju
116	Anil	152	Barra de Guaratiba	40	Bonsucesso
117	Gardênia Azul	155	Jacarezinho	57	São Francisco Xavier
12	Benfica	157	Maré	75	Vista Alegre
127	Itanhangá	158	Parque Colúmbia	92	Zumbi
129	Camorim	159	Vasco da Gama		

Table 3: Rio de Janeiro district numbers (#) and their corresponding names, for the 23 potential outliers found in Section 3.2 of the manuscript.

C Simulation study: generating data from the proposed BYM2-Gamma model

To assess the proposed BYM2-Gamma model's ability to recover the truth, a simulation study is conducted wherein data are generated from the proposed BYM2-Gamma model for 100 replicates. The $n = 160$ districts of Rio de Janeiro and their neighbourhood structure are used. The latent effects' unstructured and scaled spatially structured components are generated as follows:

$$\boldsymbol{\theta} \sim \mathcal{N}(\mathbf{0}, \boldsymbol{I}), \quad \text{and} \quad \boldsymbol{u}^{\star} \sim \mathcal{N}(\mathbf{0}, \boldsymbol{Q}_{\star}^{-}),$$

where $\boldsymbol{Q}_{\star} = h(\boldsymbol{D} - \boldsymbol{W})$, with h, the scaling factor, entirely defined by the spatial structure of Rio de Janeiro. An algorithm to generate from the ICAR prior is presented in Chapter 2 of Rue and Held [1]. The mixing components that induce the marginal heavier tails, $\boldsymbol{\kappa}$, are independently generated from a Gamma$(\nu/2, \nu/2)$, with ν fixed at 4 to allow for fairly heavy tails. The latent effects are then computed as

$$b_i = \left[\sqrt{1-\lambda}\theta_i + \sqrt{\lambda}u_i^{\star}\right] \times \sigma/\sqrt{\kappa_i}, \; i = 1, \ldots, n,$$

where $\lambda = 0.8$ and $\sigma = 0.3$. Finally, a population of size $n = 160$ is generated from the Poisson model

$$Y_i \sim \mathcal{P}\left(E_i \exp\left[\beta_0 + b_i\right]\right),$$

with $\beta_0 = -0.1$ and the offsets, $[E_1, \ldots, E_n]^{\top}$, taken from the analysis of the Zika counts. Then, models BYM2-Gamma and Congdon are fitted to each of the 100 replicates, using the same inference procedure as in section 3.1. The goal is to check if we recover the true values used to generate the data, and to check if the WAIC is able to distinguish between the proposed model and Congdon's.

Figure 2 shows that the WAIC is able to always choose the model that generated the data, namely the BYM2-Gamma model. Figure 3 presents the posterior summaries obtained from the BYM2-Gamma model across the 100 replicates, as well as box-plots of the posterior means estimated across the 100 replicates, for the intercept, β_0, the mixing parameter, λ, the hyperparameter, ν, and the overall standard deviation, σ. For all samples, the 95% posterior credible intervals of all parameters contain the true values used to generate the data. The interest lies particularly on the main parameters of the model, such as the outlier indicators, $\boldsymbol{\kappa}$. Figure 4 plots the posterior summaries, for one replicate, of the κ's across all districts in Rio de Janeiro. Most of the 95% posterior credible intervals for κ contain the true value used to generate the data. Moreover, for those neighbourhoods that have outlying observations, the estimate for κ is quite concentrated around its true value. This suggests that the model is able to point out the neighbourhoods with outlying observations. Similarly, the true latent effects, $\boldsymbol{b}$, are shown to be recovered by the 95% posterior credible intervals in Figure 5.

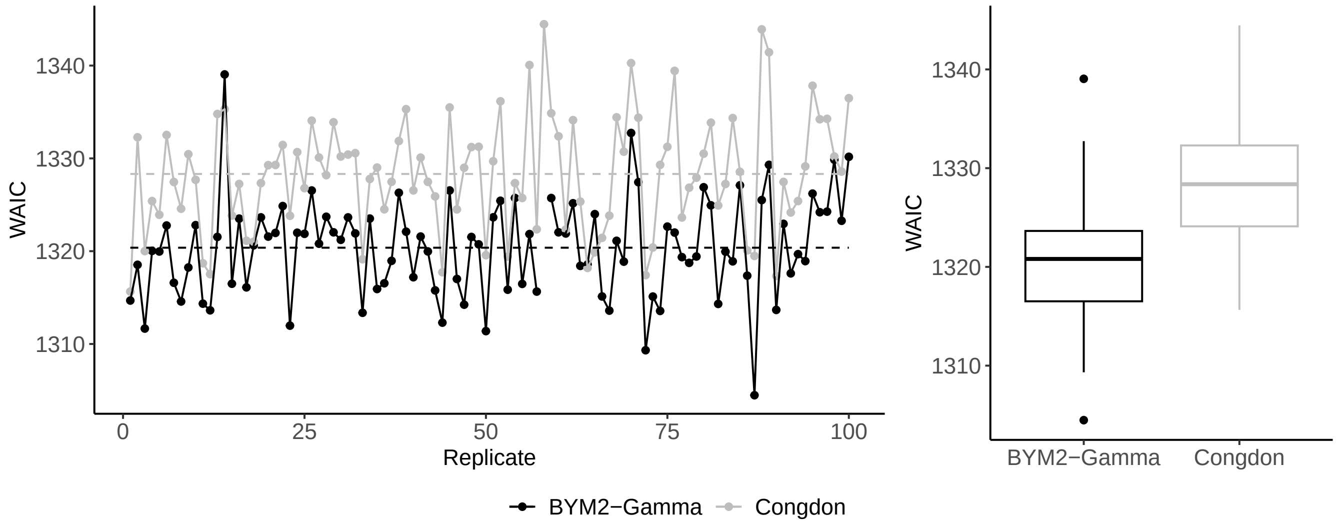

Figure 2: Left panel: WAIC across the 100 replicates for the proposed BYM2-Gamma model and Congdon's regarding the simulated data from the proposed BYM2-Gamma model. Dashed lines: mean WAIC for each model. Right panel: Box-plots of the WAIC values across the 100 simulation replicates for each model.

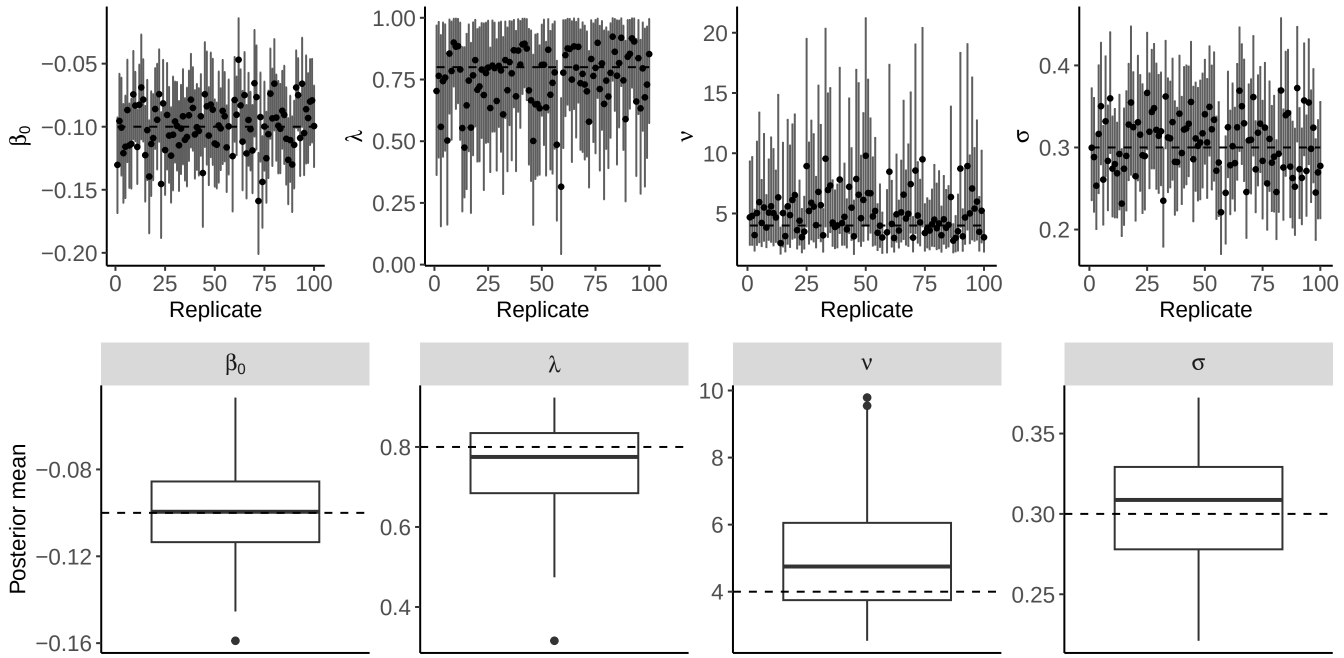

Figure 3: Top panel: Posterior summaries of the parameters for the proposed BYM2-Gamma model across the 100 replicates regarding the simulated data from the proposed BYM2-Gamma model. Solid circle: posterior mean; Vertical lines: 95% posterior credible interval. Bottom panel: Boxplots of the estimated posterior means across the 100 simulation replicates for each parameter. Dashed horizontal line: true value.

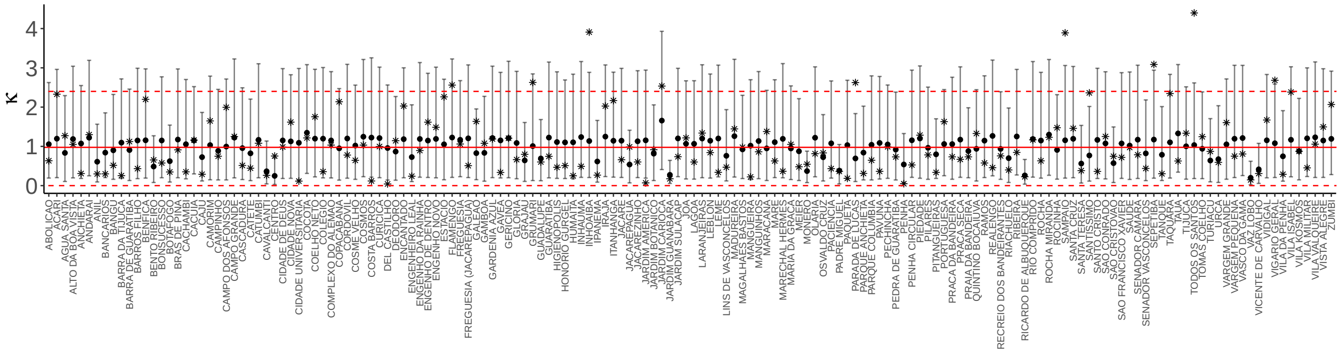

Figure 4: Posterior summaries (mean and 95% credible interval) of the κ parameters across all the districts of Rio de Janeiro for one replicate when fitting the BYM2-Gamma model. The stars correspond to the true generated κ's and the red horizontal lines correspond to the prior summary (solid line: prior mean, dashed lines: prior 95% credible interval).

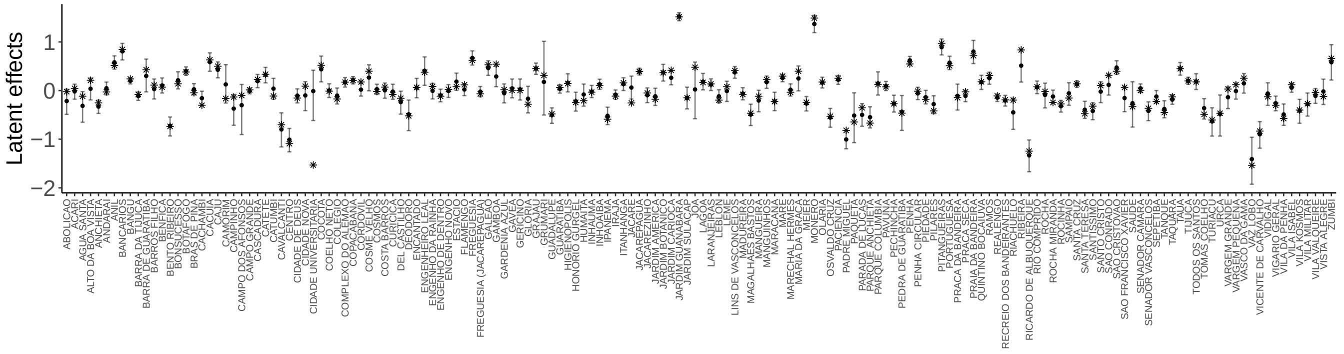

Figure 5: Posterior summaries (mean and 95% credible interval) of the latent effects across all the districts of Rio de Janeiro for one replicate when fitting the BYM2-Gamma model. The stars correspond to the true generated latent effects.

D Simulation study: generating data from the proposed BYM2-logCAR model

We now assess the proposed BYM2-logCAR model's ability to recover the truth. Similar to Appendix C, a simulation study is conducted wherein 100 replicated datasets are generated from the proposed BYM2-logCAR model using the $n = 160$ districts of Rio de Janeiro. The unstructured and spatially structured components, $\boldsymbol{\theta}$ and $\boldsymbol{u}^{\star}$ respectively, are independently generated, like in Appendix C. The scaling mixture components, $\boldsymbol{\kappa}$, are generated using the spatial structure as follows:

$$\boldsymbol{z} \mid \nu_\kappa \sim \mathcal{N}\left(\boldsymbol{0}, \nu_\kappa \boldsymbol{Q}_{\alpha,\star}^{-1}\right) \quad \text{and} \quad \kappa_i = \exp\left(-\frac{\nu_\kappa}{2} + z_i\right), \; i = 1, \ldots, n,$$

where $\boldsymbol{Q}_{\alpha,\star} = h\boldsymbol{Q}_\alpha = h_\alpha[\boldsymbol{D} - \alpha\boldsymbol{W}]$ is again the valid precision matrix that is scaled by h_α, which is computed based on $\boldsymbol{D} - \alpha\boldsymbol{W}$. We impose $\alpha = 0.99$ and define an arbitrary $\nu_\kappa = 0.3$ to allow the κ's to depart from 1. Like in Appendix C, the latent effects are then computed as $b_i = \left[\sqrt{1-\lambda}\theta_i + \sqrt{\lambda}u_i^{\star}\right] \times \sigma/\sqrt{\kappa_i}$, $i = 1, \ldots, n$, where $\lambda = 0.8$ and $\sigma = 0.3$. Finally, the population of size $n = 160$ is generated from the Poisson model, $Y_i \sim \mathcal{P}\left(E_i \exp\left[\beta_0 + b_i\right]\right)$, with $\beta_0 = -0.1$ and the offsets, $[E_1, \ldots, E_n]^\top$, taken from the analysis of the Zika counts. The proposed BYM2-logCAR model and Congdon's are both fitted on the 100 replicated datasets using the same inference procedure as in section 3.1.

Figure 6 shows that the WAIC always favours the proposed BYM2-logCAR model, which generated the data. Figure 7 shows how well the proposed BYM2-logCAR model is able to recover the true values of the model parameters through the posterior summaries across the 100 replicates and box-plots of the posterior means estimated across the 100 replicates for the intercept, β_0, the mixing parameter, λ, the hyperparameter, ν_κ, and the overall standard deviation, σ. Across the

100 replicates, the proposed BYM2-logCAR model always captures the truth, as the posterior 95% credible intervals (vertical lines) always cover the true values of the parameters (solid horizontal lines). Regarding the scaling mixture components, $\boldsymbol{\kappa}$, Figure 8 shows the posterior summaries for one replicate and generated values, across all districts. The κ's generated following this structured prior seem to vary less than the ones generated from the independent gamma priors in Appendix C. Therefore, the posterior credible intervals are narrower than the ones from the simulation study presented in Appendix C. Regardless, the posterior 95% credible intervals almost always cover the true mixture components. Similarly, the generated latent effects plotted in Figure 9 are recovered by the posterior 95% credible intervals.

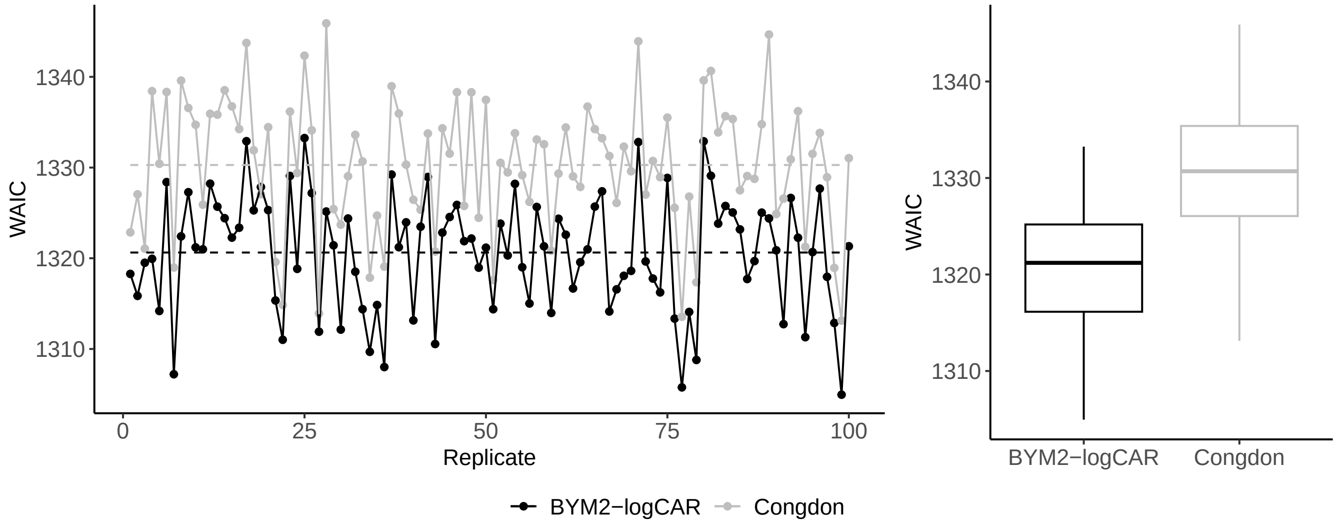

Figure 6: Left panel: WAIC across the 100 replicates for the proposed BYM2-logCAR model and Congdon's regarding the simulated data from the proposed BYM2-logCAR model. Dashed lines: mean WAIC for each model. Right panel: Box-plots of the WAIC values across the 100 simulation replicates for each model.

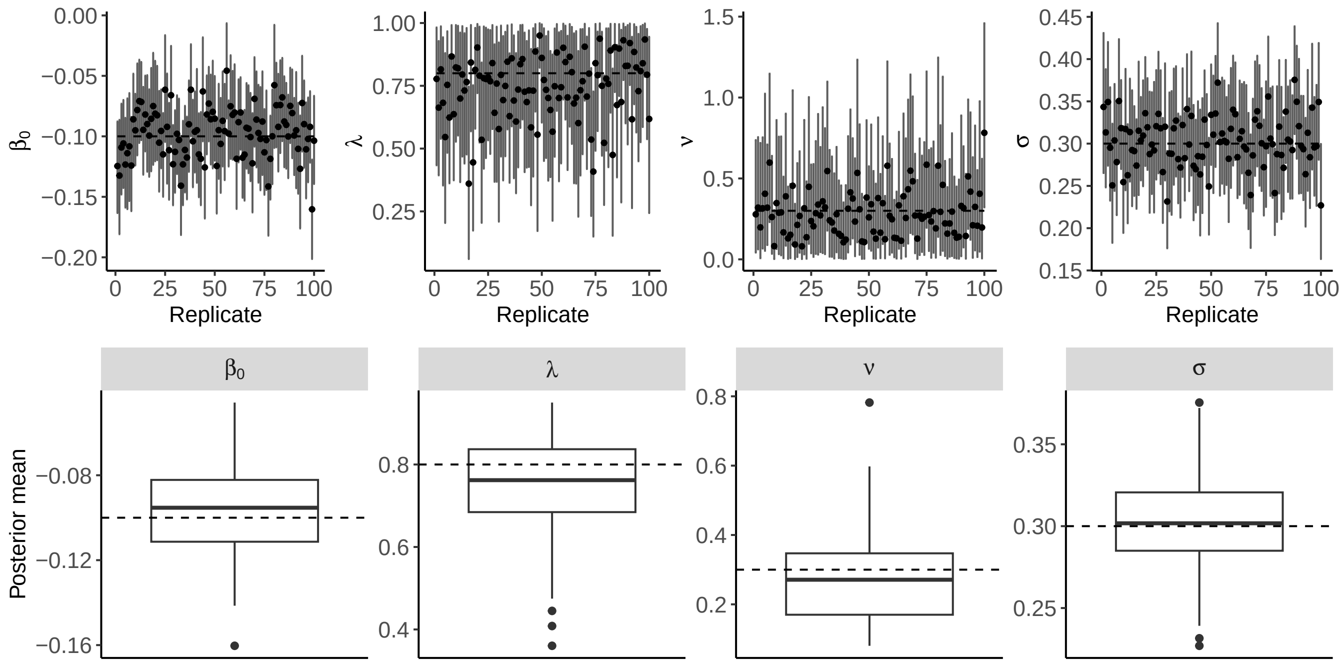

Figure 7: Top panel: Posterior summaries of the parameters for the proposed BYM2-logCAR model across the 100 replicates regarding the simulated data from the proposed BYM2-logCAR model. Solid circle: posterior mean; Vertical lines: 95% posterior credible interval. Bottom panel: Boxplots of the estimated posterior means across the 100 simulation replicates for each parameter. Dashed horizontal line: true value.

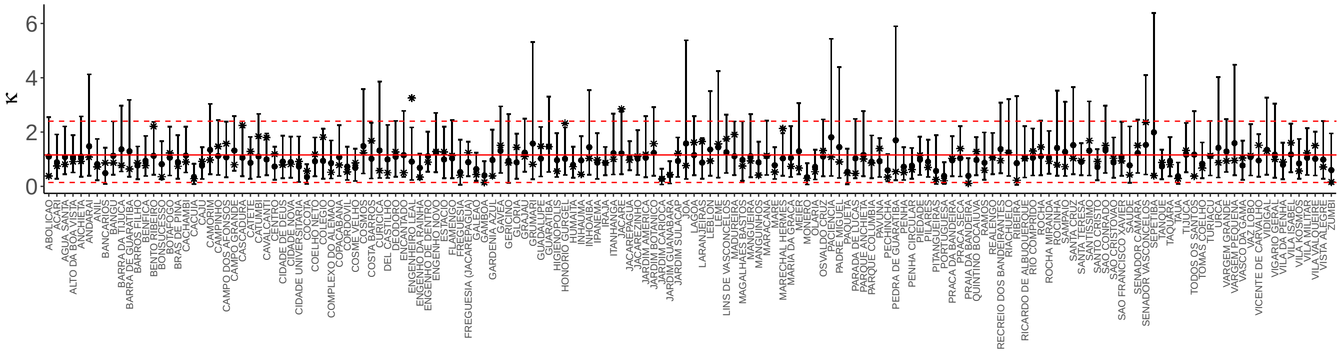

Figure 8: Posterior summaries (mean and 95% credible interval) of the κ parameters across all the districts of Rio de Janeiro for one replicate when fitting the BYM2-logCAR model. The stars correspond to the true generated κ's and the red horizontal lines correspond to the prior summary (solid line: prior mean, dashed lines: prior 95% credible interval).

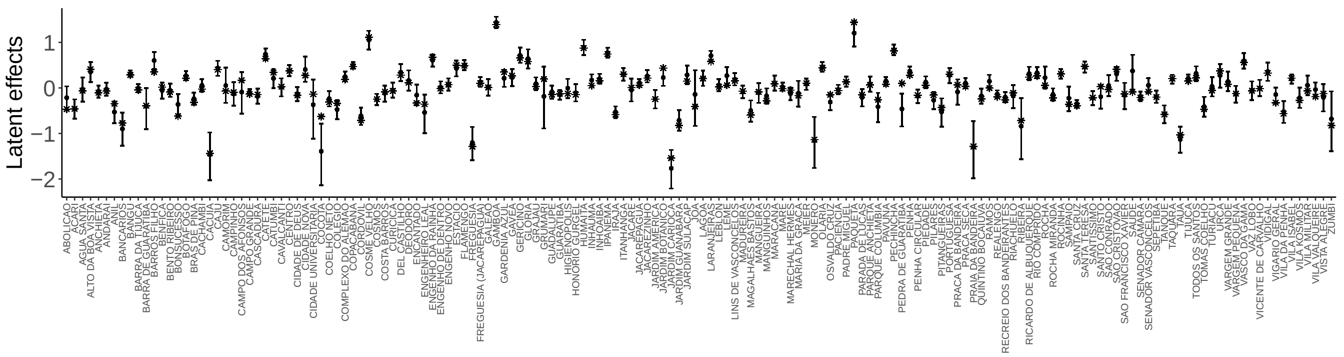

Figure 9: Posterior summaries (mean and 95% credible interval) of the latent effects across all the districts of Rio de Janeiro for one replicate when fitting the BYM2-logCAR model. The stars correspond to the true generated latent effects.

E Simulation study: no outlying areas

To confirm that the proposed model does not detect outliers when unnecessary, a simulation study is again conducted on the map of Rio de Janeiro without contaminating any district. Data are generated 100 times as follows:

$$Y_i \sim \mathcal{P}\left(E_i \exp[\beta_0 + b_i]\right),\ i = 1, \ldots, n,$$

with $n = 160$, $\beta_0 = -0.1$, $\boldsymbol{E} = [E_1, \ldots, E_n]^\top$ taken from the Zika data analysis. The latent effects, $\boldsymbol{b} = [b_1, \ldots, b_n]^\top$, are simulated once from a PCAR distribution:

$$\boldsymbol{b} \sim \mathcal{N}\left(\mathbf{0}, \sigma_b^2 \left[\boldsymbol{D} - \alpha \boldsymbol{W}\right]^{-1}\right),$$

with $\sigma_b = \sqrt{0.2}$ and $\alpha = 0.7$. Figure 10 shows the map of the 50th replicate of the simulated dataset, where no district seems to be an outlier with respect to the whole city. Again, the two parametrisations of the proposed model are compared to Congdon's, using the same prior distributions as described in section 3.1.

In terms of WAIC, the proposed models seem to perform best, as shown in Figure 11. For this simulation study, the interest lies particularly in comparing the outliers detections from the two versions of the proposed model and Congdon's. Figure 12 presents the districts that are found to be outliers by the BYM2-Gamma proposed model (a), the BYM2-logCAR proposed model (b) and Congdon's (c). The BYM2-Gamma model only identifies one district, Freguesia, to be a potential outlier in 2% of the replicates. The BYM2-logCAR and Congdon's models on the other hand detect Freguesia up to 8% of the times, showing more sensitivity to the neighbourhood structure. Congdon's model further identifies 5 districts as potential outliers although no district was contaminated.

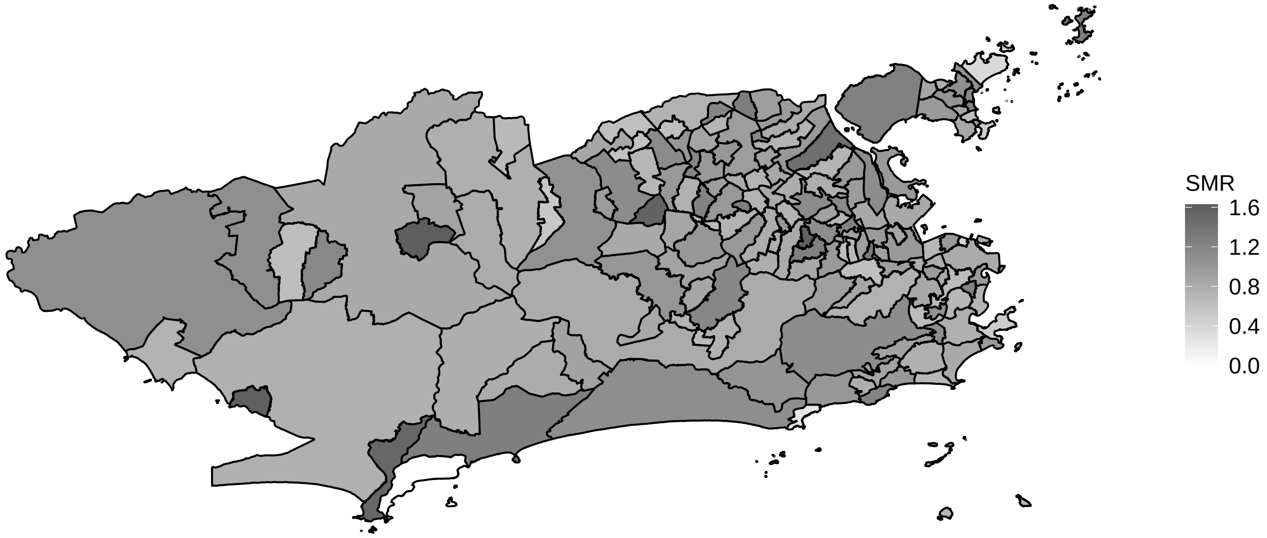

Figure 10: Standardised morbidity ratio for the 50th simulation without outliers.

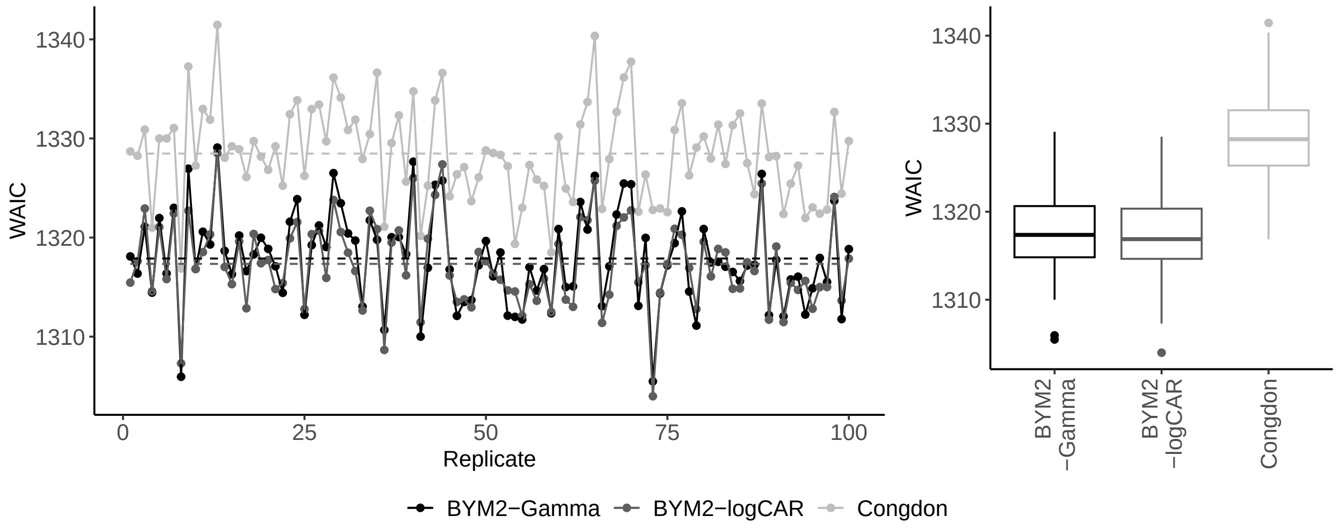

Figure 11: Left panel: WAIC across the 100 replicates for the proposed models and Congdon's for the simulation without outliers. Dashed lines: mean WAIC for each model. Right panel: Box-plots of the WAIC values across the 100 simulation replicates for each model.

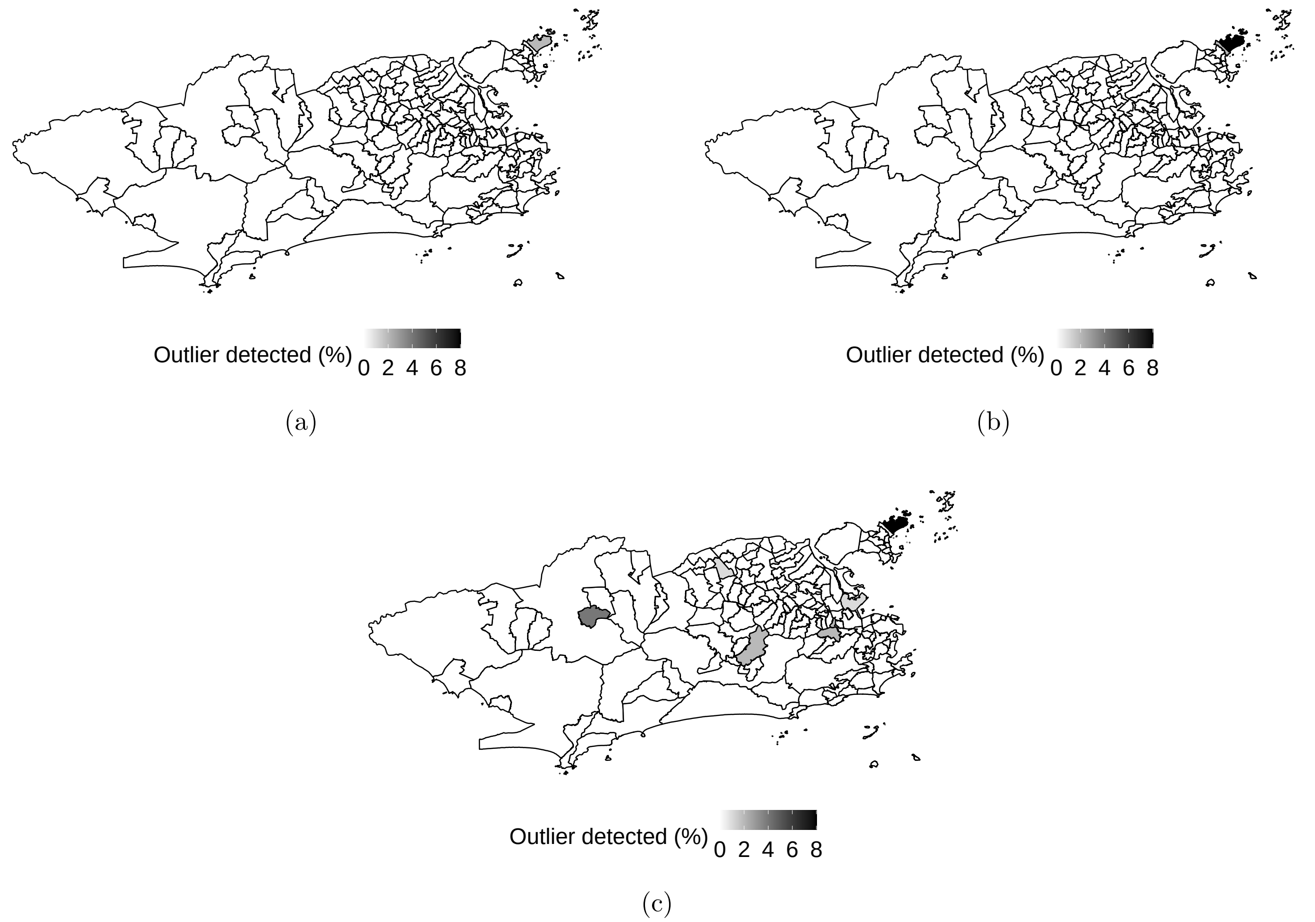

Figure 12: Maps of the percentages of outliers as indicated by $\kappa_{ur} < 1$ across the $r = 1, \dots, 100$ replicates, where κ_{ur} is the upper bound of the posterior 95% credible interval of κ in the rth replicate of the simulated dataset without outliers. a) BYM2-Gamma model; b) BYM2-logCAR model; c) Congdon's model.

F Simulation study: distant outliers in France

In this simulation study, 20 distant French departments are contaminated such that outliers are created. Similar to the simulation study presented in Section 3.1, there are no covariates in this analysis, and all areas are first imposed a relative risk of 1, $\mu_i = 1$. The same five offset categories are defined. Based on these categories, we select 20 non-neighbouring departments to be outliers. Four departments are chosen from each offset category. That is, there are 4 outliers within the smallest offset group, 4 within the second-to-smallest offset group, and so on. Then, within each group of four departments, the relative risks are contaminated into outliers by setting the relative risks to be equal to $\mu_i = 0.25$, $\mu_{i'} = 0.5$, $\mu_{i''} = 1.5$ and $\mu_{i'''} = 2$. The resulting outliers are mapped

in Figure 13, highlighting the offset sizes and imposed relative risks. Again, $R = 100$ populations of size $n = 96$ are created by generating the number of cases $Y_i \sim \mathcal{P}(E_i\mu_i)$. The same four models with priors defined in section 3.1 are fitted through `rstan`. After 20,000 iterations with a burn-in period of 10,000 and a thinning factor of 10, the 2 MCMC chains attained convergence as assessed by trace plots, effective sample sizes and $\widehat{R}$ statistics.

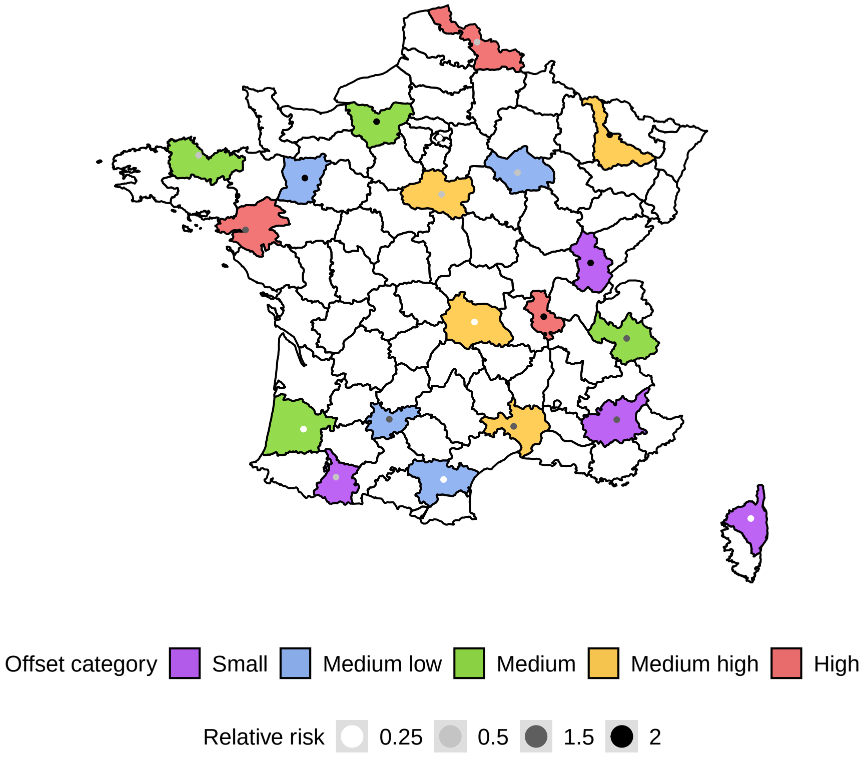

Figure 13: French departments arbitrary chosen to be outliers in the simulation study with distant outliers. Colours depict the offset category based on the empirical offset quantiles. The points represent the relative risk set to each outlying district.

In terms of WAIC [2], for which smaller values are preferred, the proposed BYM2-Gamma model performs similarly to Congdon's, as shown in Figure 14 and Table 4. The BYM2-Gamma and original Congdon models always perform better than the models that include spatially structured scaling mixture components. On average, the BYM2-logCAR and Congdon-logCAR models yield a criterion of 983, while the BYM2-Gamma and Congdon models present a WAIC of 958 and 959, respectively.

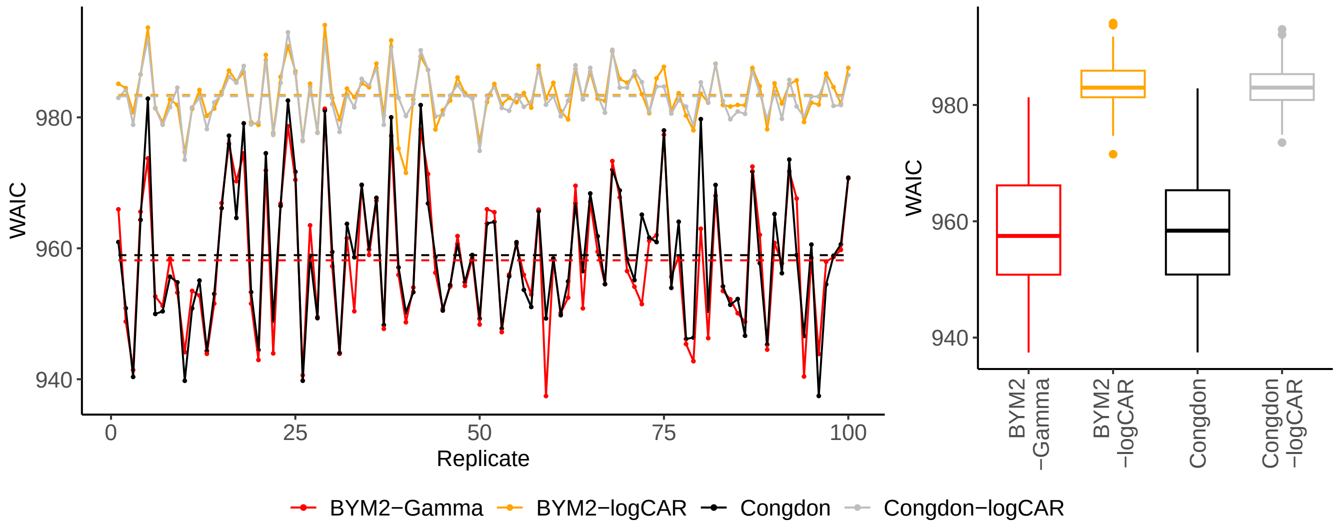

Figure 14: Left panel: WAIC across the 100 replicates for the proposed models and Congdon's, in the simulation study with distant outliers. Dashed lines: mean WAIC for each model. Right panel: Box-plots of the WAIC values across the 100 simulation replicates for each model.

The models' performances are also compared in terms of MSE, as shown in Figure 15 and Table 4. As expected, all models result in MSEs that are smaller in the areas with large offsets, and MSEs that are larger in the areas with small offsets. Additionally, all models tend to better fit the data in non-outlying areas, that is in the areas with a relative risk of 1. Regarding the outlying areas only, the largest MSEs are observed for extreme risks of 2 whereas the smallest correspond to extreme risks of 0.5. On average over the 100 replicated datasets and across all areas, the MSEs are of 0.0010 for the BYM2-Gamma and Congdon models, and 0.0011 for both log-CAR parametrisations.

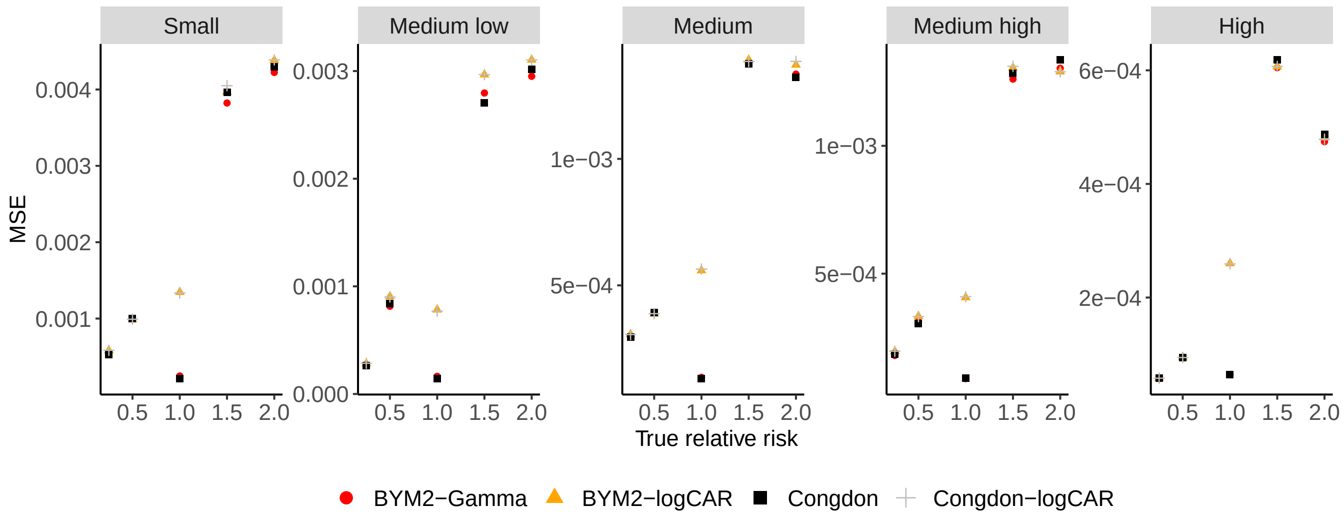

Figure 15: MSE over the 100 replicates for the proposed models and Congdon's according to the true relative risk and the offset size, in the simulation study with distant outliers.

	BYM2-Gamma	BYM2-logCAR	Congdon	Congdon-logCAR
WAIC	958.2	983.4	959.0	983.2
Mean absolute bias ×100	0.012	0.035	0.024	0.040
MSE	0.0004	0.0008	0.0004	0.0008
Coverage	98.6	96.4	98.5	96.5
Proper interval score	0.085	0.127	0.085	0.127

Table 4: Mean absolute bias multiplied by a factor of 100, MSE, coverage of the 95% posterior credible intervals, and proper interval scores of the credible intervals over the 100 replicates for the proposed and Congdon's models, in the simulation study with distant outliers.

Regarding the detection of outliers, which is the main focus of this simulation study, Table 5 shows how often each model accurately detects districts as outliers (sensitivity) and non-outliers (specificity), depending on the offset category. Additionally, Figure 16 shows how often each district is detected as a potential outlier by the four models, while indicating the offset sizes. Recall, area i is detected as an outlier when $\kappa_{u,i} < 1$, where $\kappa_{u,i}$ is the upper bound of the 95% posterior credible interval of κ_i. Overall, all models are able to find all of the contaminated districts. Additionally, except for Congdon's model with the logCAR parametrisation, none of the models tend to point out as potential outliers too many of the non-contaminated areas (specificity greater than 99%).

	Offset category	BYM2-Gamma	BYM2-logCAR	Congdon	Congdon-logCAR
Sensitivity	Small	100.0	100.0	100.0	100.0
	Medium low	100.0	100.0	100.0	100.0
	Medium	100.0	100.0	100.0	100.0
	Medium high	100.0	100.0	100.0	100.0
	High	100.0	100.0	100.0	100.0
	Overall	100.0	100.0	100.0	100.0
Specificity	Small	99.9	99.2	100.0	88.1
	Medium low	99.9	100.0	99.9	84.0
	Medium	99.8	100.0	99.9	88.7
	Medium high	99.9	99.8	99.9	79.5
	High	99.9	99.8	100.0	87.1
	Overall	99.9	99.7	99.9	85.5

Table 5: Sensitivity and specificity of the outlier detection for each model depending on the offset size, in the simulation study with distant outliers.

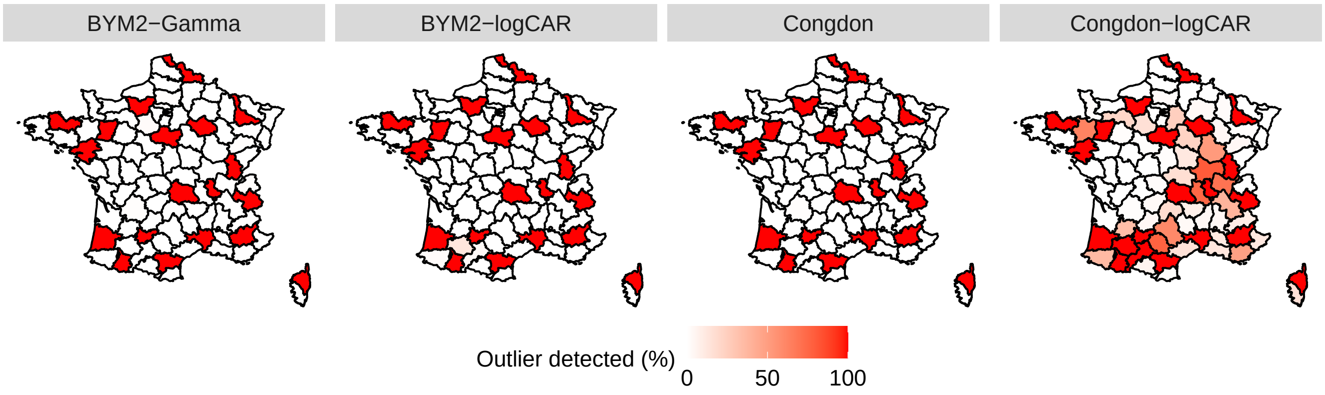

Figure 16: Percentage of times among 100 replicates that the outliers were identified by each model, in the simulation study with distant outliers. The outliers are pointed out when $\kappa_u < 1$, where κ_u is the upper bound of the posterior 95% credible interval of κ.

G Simulation studies on the map of Rio de Janeiro

In this section, we present the results from simulation studies conducted using the map of Rio de Janeiro wherein some arbitrary areas are contaminated into outlying areas, to assess the performance of the proposed model in comparison to the one proposed by Congdon[3]. Similar to Section 3.1, the design of the simulation studies is inspired by Richardson et al.[4]. The $n = 160$ districts of Rio de Janeiro and their neighbourhood structure are used as the region of study. In the first simulation study (section G.1), areas that are far from each other are contaminated into outliers. In the second simulation study (section G.2), neighbouring areas are contaminated into outliers. In the third simulation study (section G.3), neighbouring areas are contaminated and we include a covariate. In all simulation studies, the goal is to identify the correct districts as outliers.

G.1 Distant outliers in Rio

In the first simulation study, 20 districts are arbitrarily chosen to be outliers. The goal is for our proposed model to accurately identify the outliers. Out of simplicity, there are no covariates included in the generating process nor when fitting the models. First, all $n = 160$ latent effects, which correspond to log relative risks in this covariate-free simulation study, are set to 0: $b_i = 0$, $i = 1, \ldots, n$. Then, the offsets $[E_1, \ldots, E_n]^\top$ are taken from the real data application to Zika counts that is presented in section 3.2. We define five offset categories based on the empirical offset quantiles. The first category corresponds to the smallest offsets and the fifth category, to the largest ones. The categories are termed "Small" for $E \leq 59.1$, "Medium low" for $E \in (59.1, 112.4]$, "Medium" for $E \in (112.4, 177.2]$, "Medium high" for $E \in (177.2, 267.2)$ and "High" for $E > 267.2$. Based on these categories, we select 20 districts to be outliers. Four districts are chosen from each offset category. That is, there are 4 outliers within the smallest offset group, 4 within the second-to-smallest offset group, and so on. Then, within each group of four districts, the relative risks are contaminated into outliers by setting the log relative risks to be equal to $b_i = \ln(0.25)$, $b_{i'} = \ln(0.5)$, $b_{i''} = \ln(1.5)$ and $b_{i'''} = \ln(2)$. Figure 17 maps the 160 districts of Rio de Janeiro, showing which areas are outliers based on the offset category and the contaminated relative risk. Again, all the white areas have a relative risk of 1. Finally, $R = 100$ populations of size $n = 160$ are created according to a hierarchical Poisson model. That is, $Y_i \sim \mathcal{P}\left(E_i \exp[b_i]\right)$. The only source of randomness across the 100 replicates comes from the repeated sampling from a Poisson distribution.

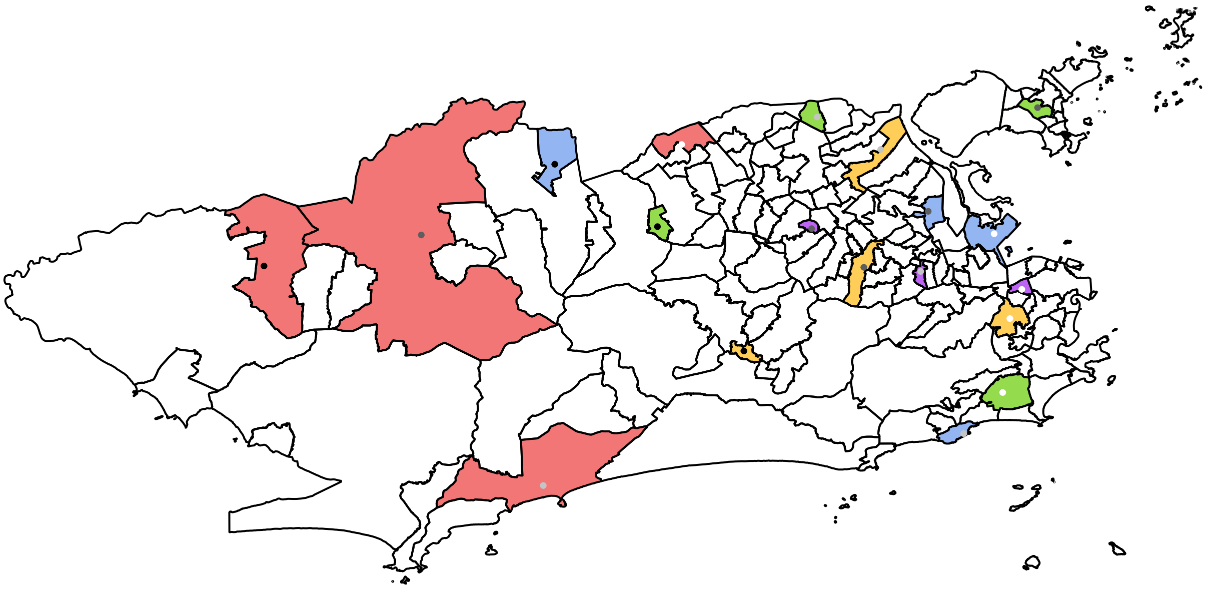

Figure 17: Districts of Rio de Janeiro city arbitrary chosen to be outliers in the simulation study with distant outliers. Colors depict the offset category based on the empirical offset quantiles. The points represent the relative risk set to each outlying district.

Using the two scale mixtures described in section 2.1, the Congdon model is compared to the proposed model. The first version of the proposed model is denoted BYM2-Gamma and the second, BYM2-logCAR. The original Congdon model is termed Congdon, whereas the one with spatially structured scale mixture components is denoted Congdon-logCAR. For the four models, the intercept is given a quite vague prior: $\beta_0 \sim \mathcal{N}(0, 10^2)$ and the mixing parameter, λ, is assigned a uniform, $\mathcal{U}(0,1)$, prior distribution. The same $\mathcal{N}_+(0,1)$ prior is considered for σ, which is a *marginal* standard deviation in the proposed model, while it is a *conditional* standard deviation in Congdon's. Finally, in the BYM2-Gamma and Congdon models, the prior distribution for the κ's is described in (5) with $\nu \sim \text{Exp}(1/4)$. For the BYM2-logCAR and Congdon-logCAR parametrisations, the κ's follow *a priori* the distribution in (5) and we set $\nu \sim \text{Exp}(1/0.3)$.

The models are fitted through the R package `rstan` (Stan Development Team [5]). For each dataset, the MCMC procedure consists of 2 chains of 20,000 iterations with a 10,000 burn-in period and a thinning factor of 10. Convergence of the chains is assessed through trace plots, effective sample sizes and the $\widehat{R}$ statistic (Gelman et al. [6], Vehtari et al. [7]).

In terms of WAIC, the proposed BYM2-Gamma model performs better than Congdon's, on

average, as shown in Figure 18. The BYM2-Gamma and original Congdon models always perform better than the models that include spatially structured scaling mixture components. On average, the BYM2-logCAR model yields a criterion of 1289.5 versus 1288.6 for the Congdon-logCAR model, while the BYM2-Gamma model presents a WAIC of 1260.9, versus 1263.9 for Congdon's.

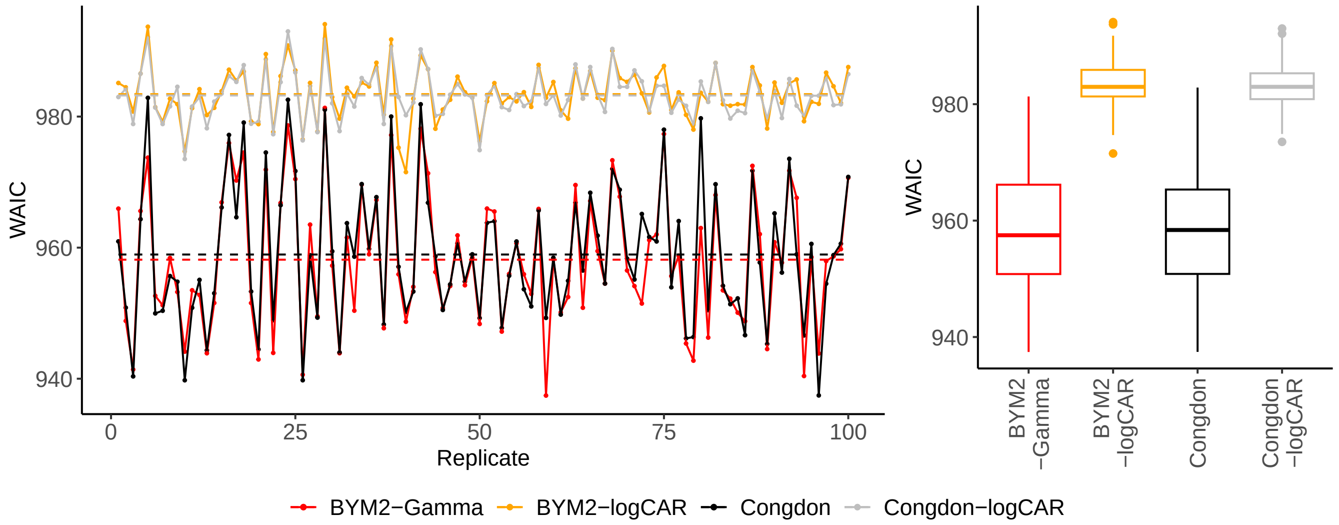

Figure 18: Left panel: WAIC across the 100 replicates for the proposed models and Congdon's in the simulation study with distant outliers in Rio de Janeiro. Dashed lines: mean WAIC for each model. Right panel: Box-plots of the WAIC values across the 100 simulation replicates for each model.

The models' performances are also compared in terms of MSE, as shown in Figure 19. Again, as expected, all models yield smaller MSEs in areas with larger offsets. Additionally, all models tend to better fit the data in areas that are not outliers, that is in the areas with a relative risk of 1. On average over the 100 replicated datasets and across all areas, the MSEs are of 0.005 for the BYM2-Gamma model, 0.006 for Congdon and 0.008 for both log-CAR parametrisations.

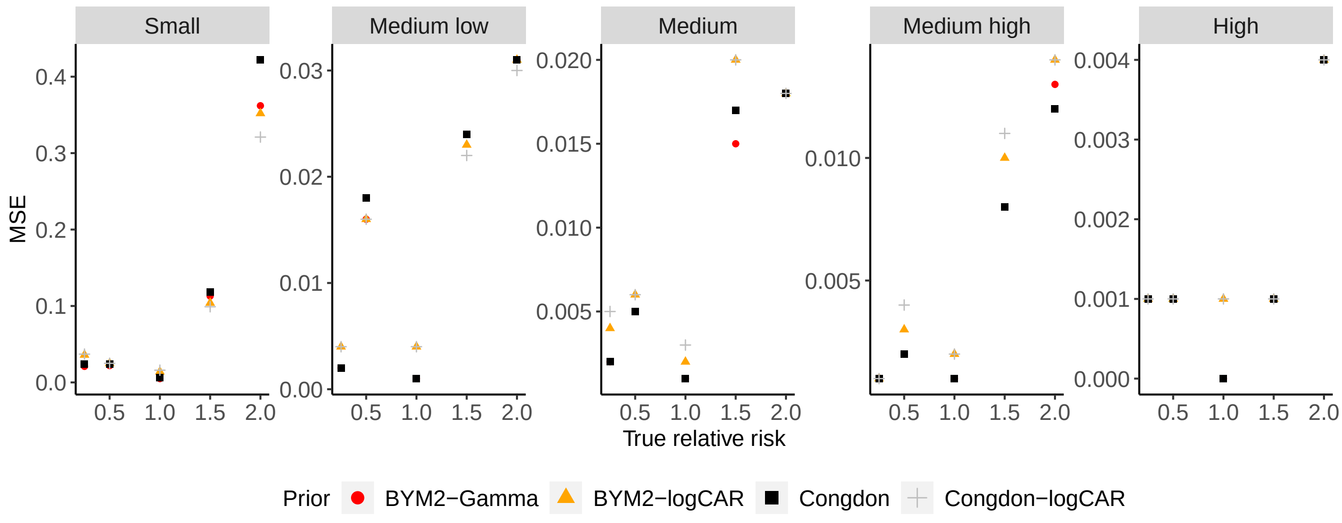

Figure 19: MSE over the 100 replicates for the proposed models and Congdon's according to the true relative risk and the offset size in the simulation study with distant outliers in Rio de Janeiro.

Regarding the detection of outliers, which is the main focus of this simulation study, Table 6 shows how often each model accurately detects districts as outliers (sensitivity) and non-outliers (specificity), depending on the offset category. That is, the sensitivity is equal to the percentage of outliers detected among the contaminated districts over the 100 replicates. The specificity is the percentage of districts not identified as outliers among the ones whose true relative risk is equal to 1, over the 100 replicates. Additionally, Figure 20 shows how often each district is detected as a potential outlier by the four models, while indicating the offset sizes. Area i is detected as an outlier when $\kappa_{u,i} < 1$, where $\kappa_{u,i}$ is the upper bound of the 95% posterior credible interval of κ_i. Overall, all models are able to find the contaminated districts in the four upper offset categories. When the offsets are the smallest, all models detect the outliers only half of the time, with a slight advantage for the proposed models (e.g. sensitivity of 55.5 for BYM2-Gamma versus 50.25 for Congdon). In this simulation study where outliers are distant, the parametrisations with spatially structured scaling mixture components tend to identify slightly more outliers than are truly present in the data (e.g. specificities of 95.4 versus 90.2 for BYM2-logCAR and Congdon-logCAR, respectively).

	Offset category	BYM2-Gamma	BYM2-logCAR	Congdon	Congdon-logCAR
Sensitivity	Small	55.50	54.00	50.25	49.50
	Medium low	94.25	94.75	91.50	94.75
	Medium	99.50	94.75	98.50	95.00
	Medium high	100.00	99.00	100.00	99.50
	High	100.00	100.00	100.00	98.50
	Overall	89.85	88.50	88.05	87.45
Specificity	Small	99.93	97.82	99.93	95.04
	Medium low	100.00	95.39	100.00	90.21
	Medium	100.00	99.64	100.00	98.93
	Medium high	99.93	99.57	99.93	98.11
	High	99.96	99.89	99.96	99.25
	Overall	99.96	98.46	99.96	96.31

Table 6: Sensitivity and specificity of the outlier detection for each model depending on the offset size, in the simulation study with distant outliers in Rio de Janeiro.

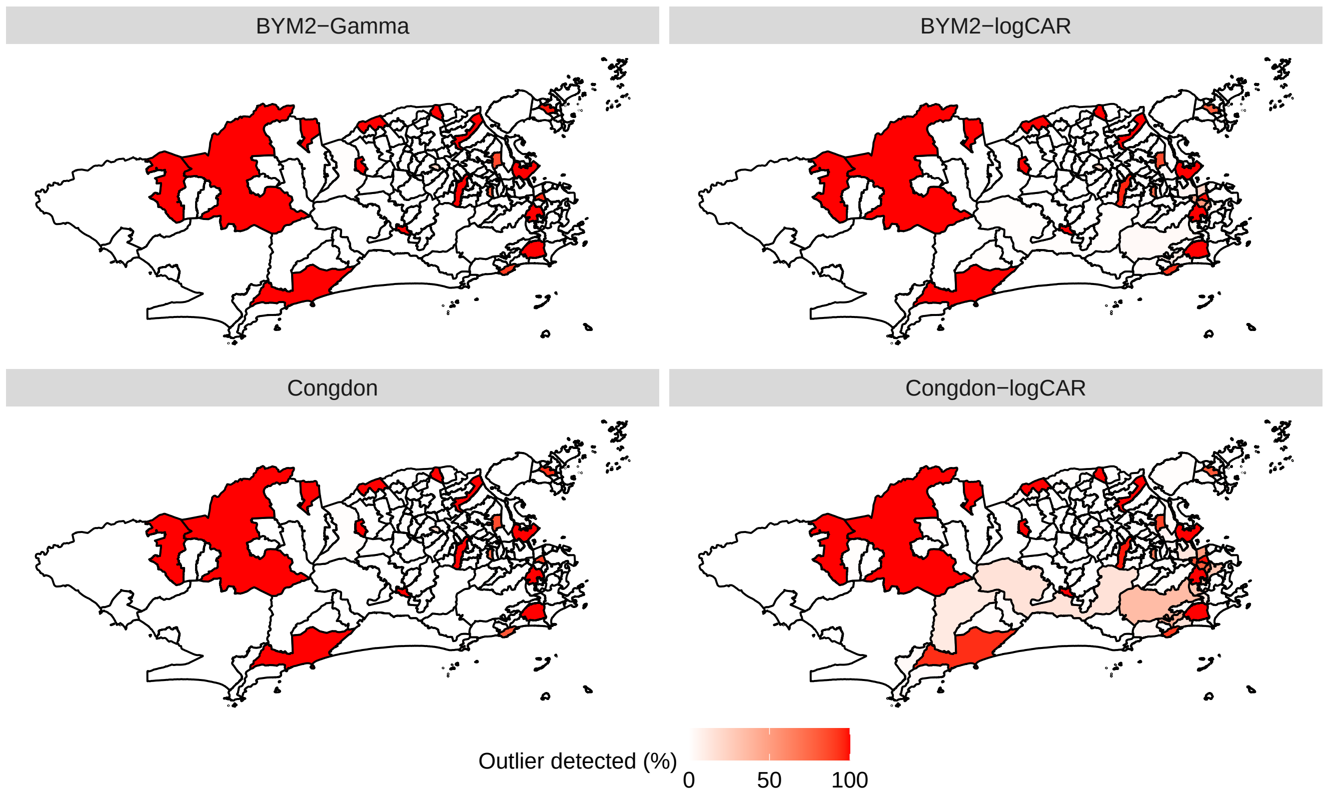

Figure 20: Percentage of times among 100 replicates that the outliers were identified by each model, in the simulation study with distant outliers in Rio de Janeiro. The outliers are pointed out when $\kappa_u < 1$, where κ_u is the upper bound of the posterior 95% credible interval of κ.

G.2 Neighbouring outliers in Rio

In this second simulation study using the map of Rio de Janeiro, 20 districts are contaminated such that 2 groups of 10 neighbouring outliers are created. Once again, there are no covariates in this analysis and all areas are first imposed a relative risk of 1. Similarly to section G.1, the offsets $[E_1, \ldots, E_n]^\top$ are taken from the Zika data analysis from section 3.2. Hence, the same five offset categories are defined. Then, 20 districts are selected to be outliers, such that each group of 10 neighbouring outliers contains 2 areas of each offset category. Within each such pair of districts, the relative risks are contaminated into outliers by setting $b_i = \ln(0.5)$ and $b_{i'} = \ln(1.5)$. The resulting outliers are mapped in Figure 21, highlighting the offset sizes and imposed relative risks. Again, $R = 100$ populations of size $n = 160$ are created by generating the number of cases $Y_i \sim \mathcal{P}\left(E_i \exp[b_i]\right)$. The same four models with priors defined in section 3.1 are fitted through `rstan`. After 20,000 iterations with a burn-in period of 10,000 and a thinning factor of 10, the 2 MCMC chains attained convergence as assessed by trace plots, effective sample sizes and $\widehat{R}$ statistics.

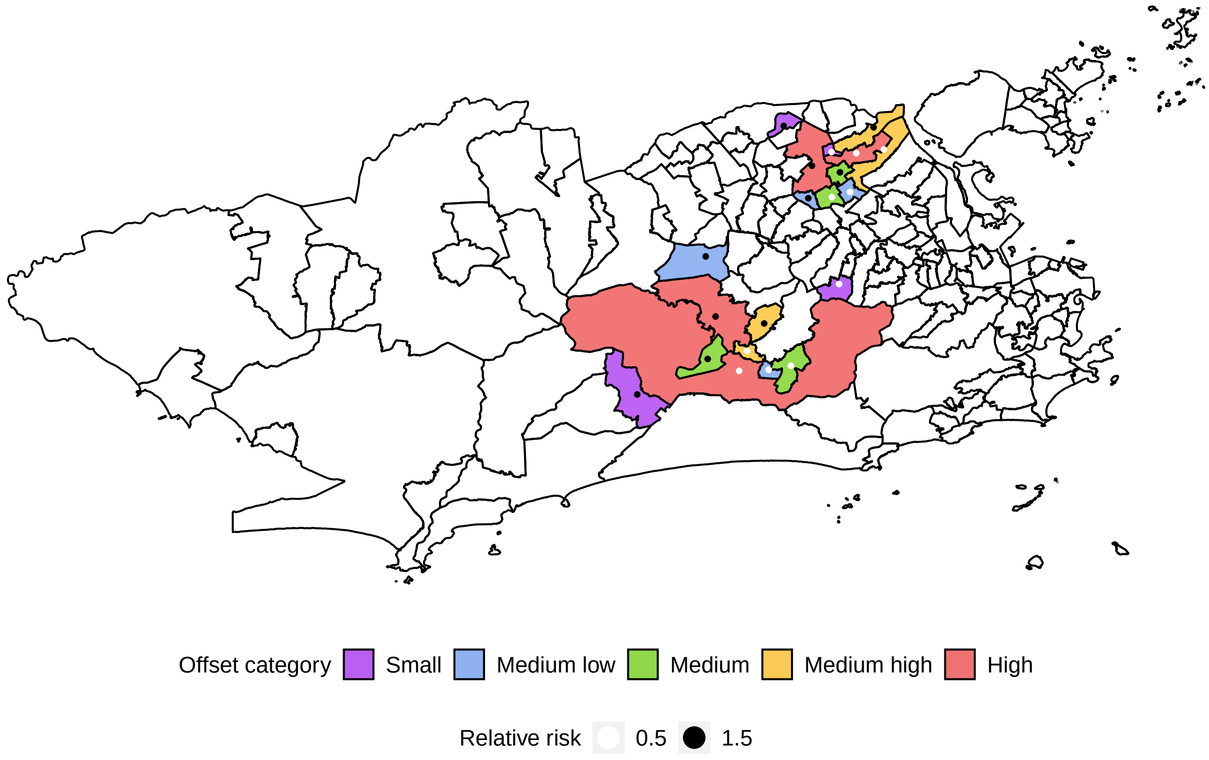

Figure 21: Districts of Rio de Janeiro city arbitrary chosen to be outliers in the simulation study with neighbouring outliers. Colors depict the offset category based on the empirical offset quantiles. The points represent the relative risk set to each outlying district.

In terms of WAIC, as shown in Figure 22, Congdon performs slightly worse than the other three models, with an average value of 1275, versus 1270 for Congdon-logCAR the two proposals.

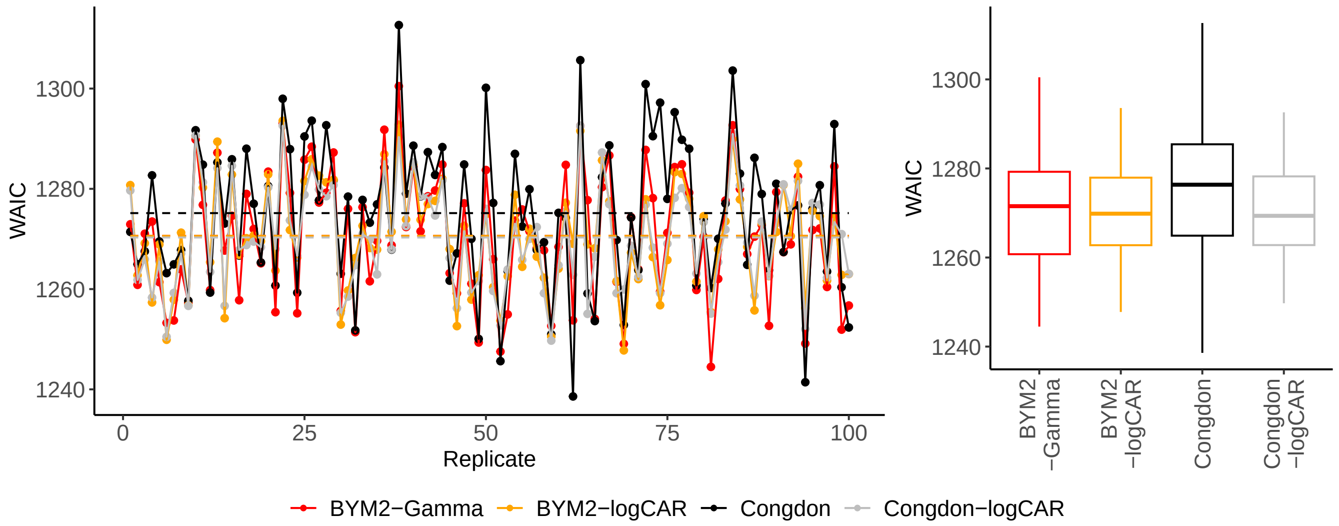

Figure 22: Left panel: WAIC across the 100 replicates for the proposed models and Congdon's in the simulation study with neighbouring outliers in Rio de Janeiro. Dashed lines: mean WAIC for each model. Right panel: Box-plots of the WAIC values across the 100 simulation replicates for each model.

In terms of MSE, as expected, all models fit better the data in areas with higher offsets than in areas with smaller offsets, as shown in Figure 23. Again, all models better fit the data in areas that are not outliers, areas with a relative risk of 1. Over the 100 replicates and all areas, the four models perform similarly, with an average MSE of 0.004.

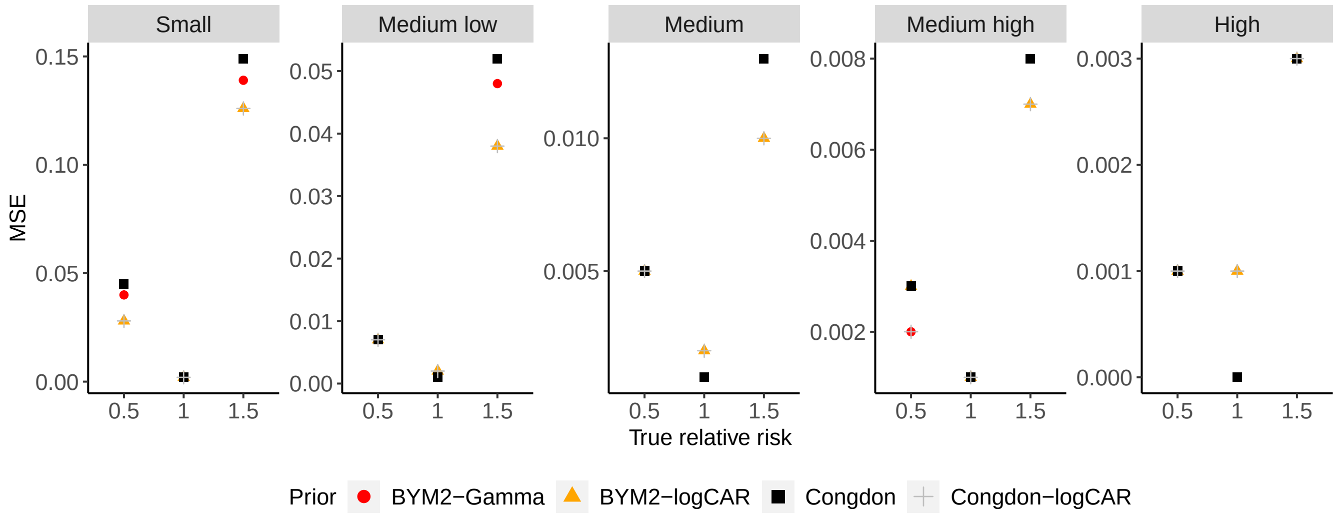

Figure 23: MSE over the 100 replicates for the proposed models and Congdon's according to the true relative risk and the offset size, in the simulation study with neighbouring outliers in Rio de Janeiro.

Regarding the detection of outliers, the results are summarised in Table 7 and Figure 24. Similarly to the previous simulation study with distant outliers, both models with spatially structured κ's tend to identify more outliers than truly present in the data (e.g. overall specificities of 97% and 96.5% for BYM2-logCAR and Congdon-logCAR, respectively, versus 99.9% for both BYM2-Gamma and Congdon). In the smallest offset category, all models often miss the outliers, with a clear advantage for the models with spatially structured κ's (e.g. sensitivity of about 30% for BYM2-Gamma and Congdon versus 64% for BYM2-logCAR and Congdon-logCAR). Regardless of the offset size, the BYM2-Gamma model performs better than Congdon's in terms of detected outliers. In particular, in the third offset category, the BYM2-Gamma model misses outliers only 1.5% of the time versus 18.75% for Congdon's model.

	Offset category	BYM2-Gamma	BYM2-logCAR	Congdon	Congdon-logCAR
Sensitivity	Small	35.25	64.25	30.50	64.00
	Medium low	80.25	93.00	64.25	89.00
	Medium	98.50	100.00	81.25	96.25
	Medium high	100.00	100.00	91.00	100.00
	High	100.00	100.00	93.75	97.75
	Overall	82.80	91.45	72.15	89.40
Specificity	Small	100.00	100.00	99.93	100.00
	Medium low	99.96	98.07	99.96	98.00
	Medium	99.89	93.79	99.93	92.50
	Medium high	99.96	96.50	100.00	95.79
	High	99.96	96.36	99.71	96.21
	Overall	99.96	96.96	99.91	96.51

Table 7: Sensitivity and specificity of the outlier detection for each model depending on the offset size, in the simulation study with neighbouring outliers in Rio de Janeiro.

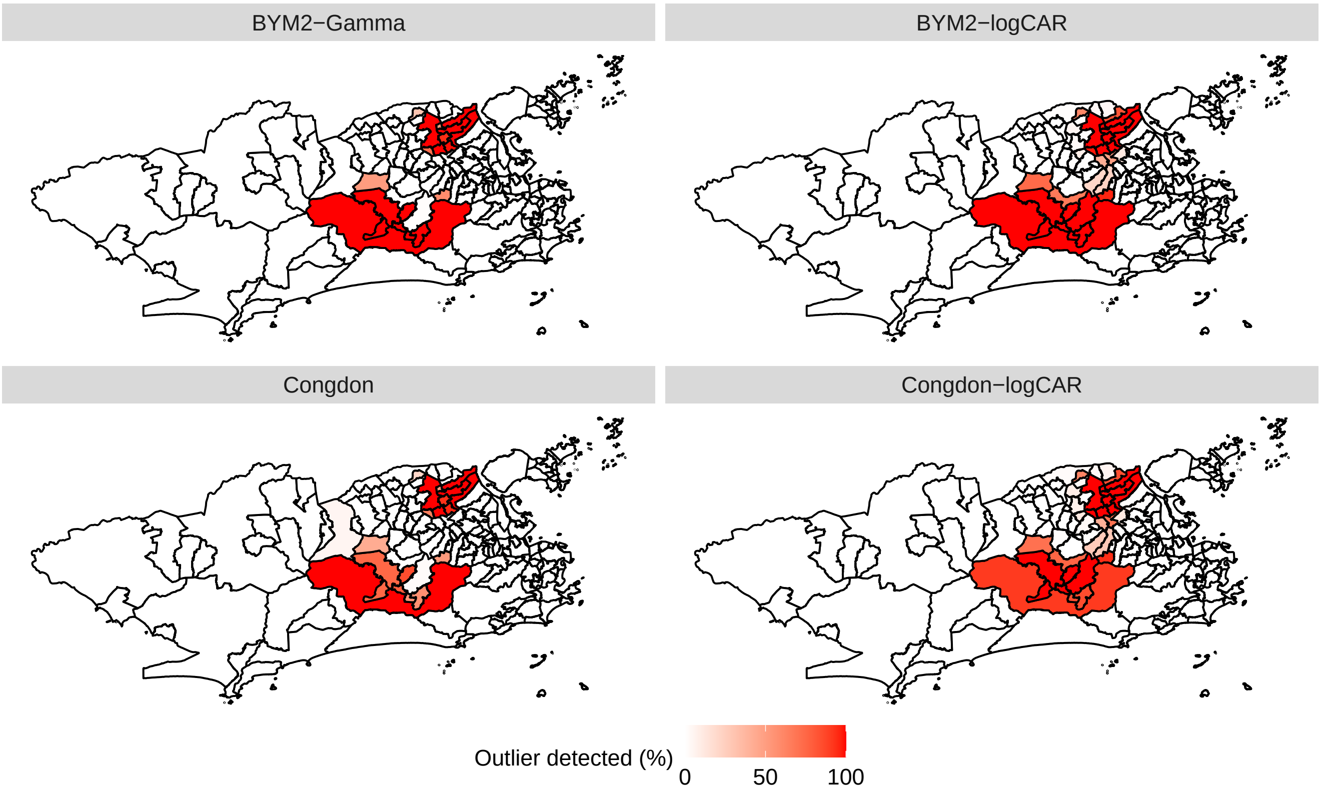

Figure 24: Percentage of times among 100 replicates that the outliers were identified by each model, in the simulation study with neighbouring outliers in Rio de Janeiro. The outliers are pointed out when $\kappa_u < 1$, where κ_u is the upper bound of the posterior 95% credible interval of κ.

G.3 Neighbouring outliers with a covariate in Rio

In this third simulation study using the map of Rio de Janeiro, the same offset categories and 2 groups of 10 neighbouring outliers as in section G.2 are chosen. Again, the goal is to identify the outlying areas. First, all $n = 160$ latent effects are generated following a proper CAR (PCAR) distribution: $\boldsymbol{b} \sim \mathcal{N}\left(\boldsymbol{0}, \sigma^2 \left[\boldsymbol{D} - \alpha \boldsymbol{W}\right]^{-1}\right)$, where the matrices $\boldsymbol{W}$ and $\boldsymbol{D}$ are computed as defined in Section 1.1, using the neighbourhood structure of Rio de Janeiro. We set $\sigma^2 = 0.1$ and $\alpha = 0.99$ such that the proper spatial distribution is close to an ICAR distribution. Following Section G.2, four districts are chosen from each offset category and their generated latent effects are contaminated as $b_i^{contam} = b_i + e_i$, with $e_i \sim \mathcal{U}\left(2\max(|b_{(1)}|, |b_{(n)}|), 3\max(|b_{(1)}|, |b_{(n)}|)\right)$, where $b_{(1)}$ and $b_{(n)}$ denote the minimum and maximum generated latent effects, respectively. Figure 25 (a) maps the resulting 160 latent effects, showing which areas are outliers based on the offset category. Finally, $R = 100$ populations of size $n = 160$ are created according to the hierarchical Poisson model $Y_i \sim \mathcal{P}\left(E_i \exp[\beta_0 + \beta x_i + b_i]\right)$, where $\beta_0 = 2.5$, $\beta = -3.5$ and the covariate x is the development index taken from the real data application to Zika counts presented in Section 3.2. The resulting relative

risks are mapped in Figure 25 (b), showing the outlying areas based on the offset category. Once again, the same four models are fitted through `rstan` and convergence of the 2 MCMC chains was attained after 20,000 iterations with a burn-in period of 10,000 and a thinning factor of 10.

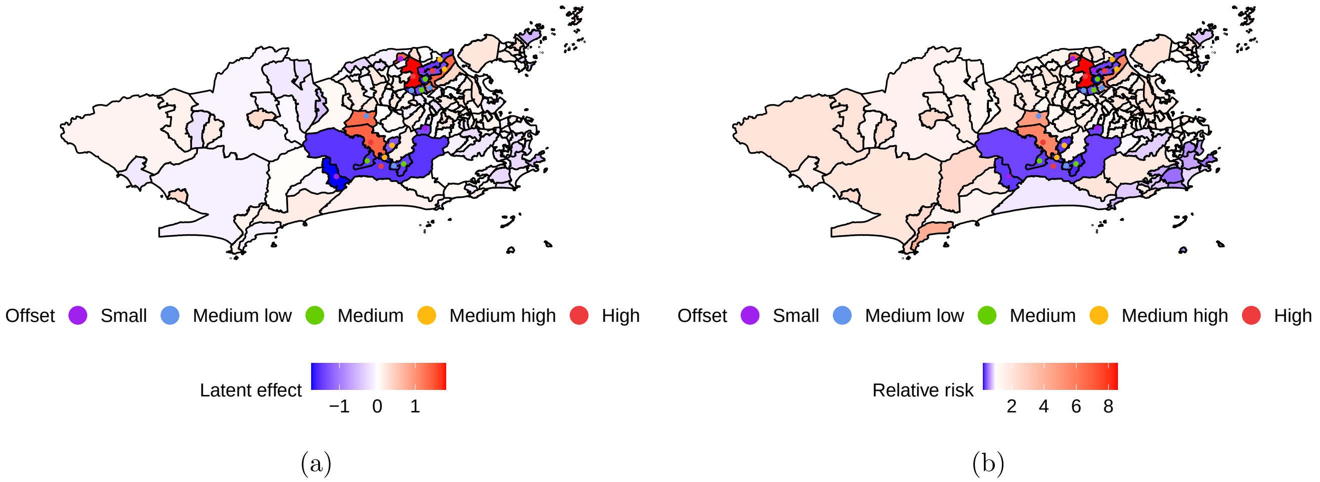

Figure 25: Rio de Janeiro maps of the latent effects (a) and relative risks (b) after contamination, in the simulation study with a covariate and neighbouring outliers. The coloured points depict the offset category based on the empirical offset quantiles.

In terms of WAIC, the proposed BYM2-Gamma model performed the best, with a mean WAIC of 1383 over the 100 replicates. As shown in Figure 26 and Table 8, the other three models' performances are similar to each other, with average values of 1389 (Congdon), 1390 (BYM2-logCAR) and 1388 (Congdon-logCAR).

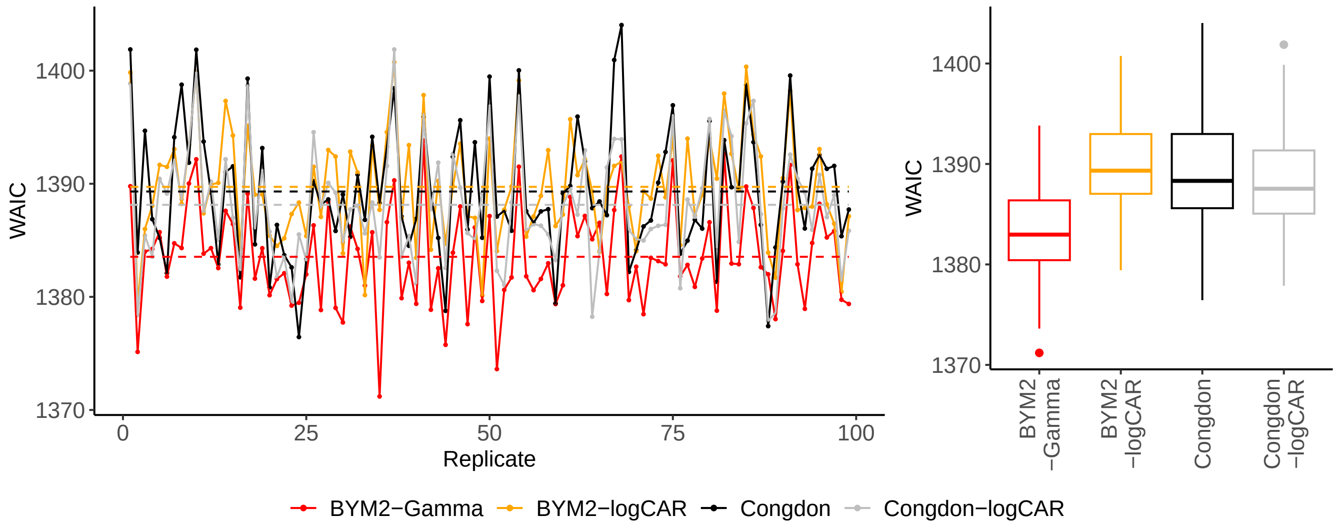

Figure 26: Left panel: WAIC across the 100 replicates for the proposed models and Congdon's, in the simulation study with a covariate and neighbouring outliers in Rio de Janeiro. Dashed lines: mean WAIC for each model. Right panel: Box-plots of the WAIC values across the 100 simulation replicates for each model.

Figure 27 shows each model's MSE for every districts across the different offset categories. The four models yield again smaller MSEs in districts with relative risks closer to 1, regardless of the offset size. Additionally, regardless of the relative risk size, all models reach smaller MSEs values for larger offset values. Overall, as shown in Table 8, the proposed BYM2-Gamma model performed better with a mean MSE of 0.0189, versus 0.0212, 0.0203 and 0.0202, for the proposed BYM2-logCAR, Congdon and Congdon-logCAR models, respectively.

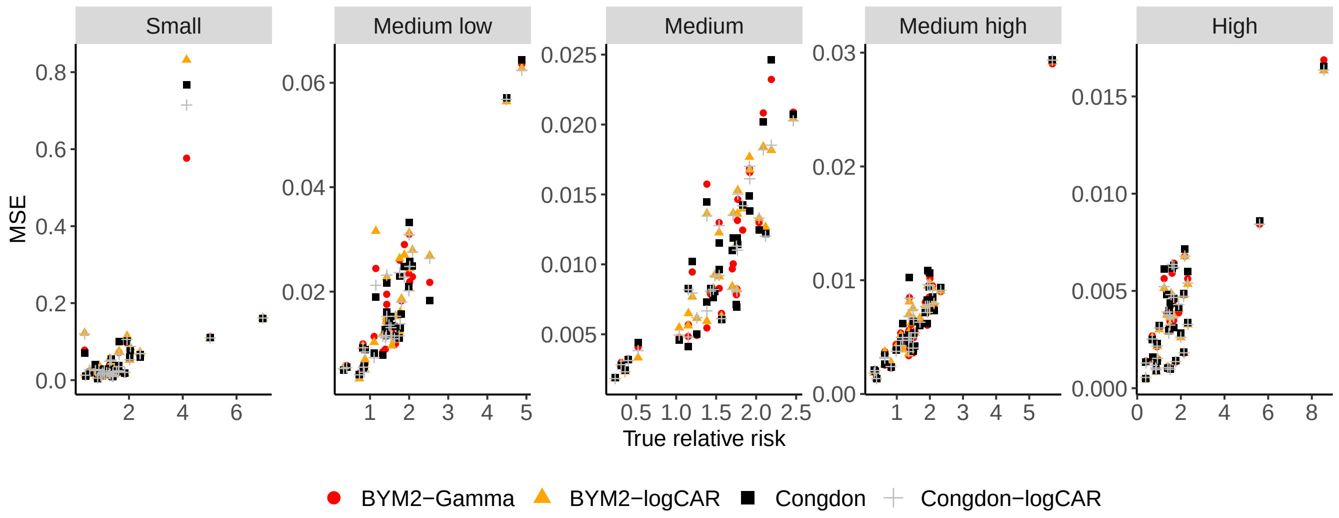

Figure 27: MSE over the 100 replicates for the proposed models and Congdon's according to the true relative risk and the offset size, in the simulation study with a covariate and neighbouring outliers in Rio de Janeiro.

	BYM2-Gamma	BYM2-logCAR	Congdon	Congdon-logCAR
WAIC	1383.5	1389.7	1389.2	1388.1
Mean absolute bias $\times 100$	0.141	0.239	0.154	0.170
MSE	0.0189	0.0212	0.0203	0.0202
Coverage	94.9	94.6	94.4	94.6
Proper interval score	0.536	0.552	0.539	0.545

Table 8: Mean absolute bias multiplied by a factor of 100, MSE, coverage of the 95% posterior credible intervals, and proper interval scores of the credible intervals over the 100 replicates for the proposed and Congdon's models, in the simulation study with a covariates and neighbouring outiers in Rio de Janeiro.

Table 9 shows the sensitivities and specificities of outlier identification produced by each model across the five offset categories. The proposed BYM2-Gamma model performs better in both identifying the correct outliers, and not pointing out the non-contaminated areas. Overall, Congdon's model misses some outlying districts 8% of the time, and up to 19% of the time, in the fourth offset category. The proposed spatially structured prior for the mixture components improved Congdon's model performance, where Congdon-logCAR only misses 2% of the contaminated areas, overall. Additionally, Congdon's model tends to capture more outliers than were contaminated, like the western and eastern non-contaminated districts that are detected 75% of the time, as shown in Figure 28.

	Offset category	BYM2-Gamma	BYM2-logCAR	Congdon	Congdon-logCAR
Sensitivity	Small	98.5	96.0	99.2	97.0
	Medium low	100.0	99.2	90.4	99.0
	Medium	100.0	99.8	90.9	99.0
	Medium high	100.0	99.5	81.8	99.5
	High	100.0	99.8	100.0	99.0
	Overall	99.7	98.8	92.5	98.7
Specificity	Small	99.3	99.9	99.1	99.9
	Medium low	98.4	99.5	96.9	98.5
	Medium	99.4	98.2	95.6	96.8
	Medium high	99.8	99.9	99.4	99.9
	High	98.3	96.3	97.9	96.3
	Overall	99.0	98.8	97.8	98.3

Table 9: Sensitivity and specificity of the outlier detection for each model depending on the offset size, in the simulation study with a covariate and neighbouring outliers in Rio de Janeiro.

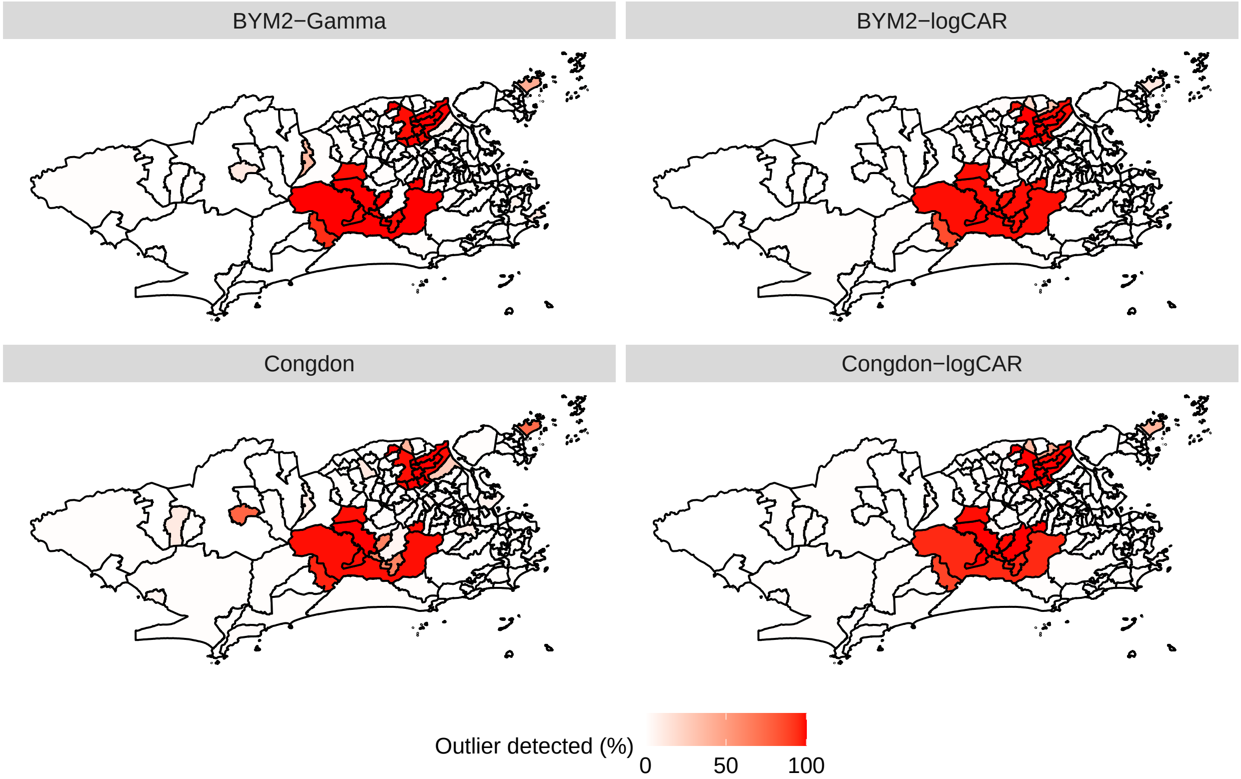

Figure 28: Percentage of times among 100 replicates that the outliers were identified by each model, in the simulation study with a covariate and neighbouring outliers in Rio de Janeiro. The outliers are pointed out when $\kappa_u < 1$, where κ_u is the upper bound of the posterior 95% credible interval of κ.

H Comparison with the model proposed by Copras-Burgos and Martinez-Beneito[8]

Table 10 compares the definitions of the proposed model, and the ones proposed by Congdon[3] and Copras-Burgos and Martinez-Beneito[8] (CB-MB). Further, Table 10 provides a comparison of the models depending on the spatial dependence parameter, λ.

Model definitions	
Congdon	$b_i \mid \boldsymbol{b}_{(-i)} \sim \mathcal{N}\left(\dfrac{\lambda}{1-\lambda+\lambda\left(\sum_{j=1}^{n} w_{ij}\right)}\sum_{j=1}^{n} w_{ij}\kappa_j b_j, \dfrac{\sigma^2}{\kappa_i\left[1-\lambda+\lambda\left(\sum_{j=1}^{n} w_{ij}\right)\right]}\right)$
CB-MB	$b_i \mid \boldsymbol{b}_{(-i)} \sim \mathcal{N}\left(\dfrac{\lambda}{1-\lambda+\lambda\left(\sum_{j=1}^{n} w_{ij}\sqrt{c_j}\right)}\sum_{j=1}^{n} w_{ij}\sqrt{c_j} b_j, \dfrac{\sigma^2}{\sqrt{c_i}\left[1-\lambda+\lambda\left(\sum_{j=1}^{n} w_{ij}\sqrt{c_j}\right)\right]}\right)$
Our Proposal	$b_i = \sigma/\sqrt{\kappa_i}\left(\sqrt{1-\lambda}\theta_i + \sqrt{\lambda/h}u_i\right)$

	$\lambda = 0$	$\lambda = 1$
Congdon	$b_i \overset{i.i.d.}{\sim} \mathcal{N}(0, \sigma^2/\kappa_i)$	$b_i \mid \boldsymbol{b}_{(-i)} \sim \mathcal{N}\left(\dfrac{1}{\left(\sum_{j=1}^{n} w_{ij}\right)}\sum_{j=1}^{n} w_{ij}\kappa_j b_j, \dfrac{\sigma^2}{\kappa_i\left(\sum_{j=1}^{n} w_{ij}\right)}\right)$
CB-MB	$b_i \overset{i.i.d.}{\sim} \mathcal{N}\left(0, \sigma^2/\sqrt{c_i}\right)$	$b_i \mid \boldsymbol{b}_{(-i)} \sim \mathcal{N}\left(\dfrac{1}{\left(\sum_{j=1}^{n} w_{ij}\sqrt{c_j}\right)}\sum_{j=1}^{n} w_{ij}\sqrt{c_j} b_j, \dfrac{\sigma^2}{\sqrt{c_i}\left(\sum_{j=1}^{n} w_{ij}\sqrt{c_j}\right)}\right)$
Our Proposal	$b_i \overset{i.i.d.}{\sim} \mathcal{N}(0, \sigma^2/\kappa_i)$	$b_i \mid \boldsymbol{b}_{(-i)} \sim \mathcal{N}\left(\dfrac{1}{\left(\sum_{j=1}^{n} w_{ij}\right)}\sum_{j=1}^{n} w_{ij}\sqrt{\dfrac{\kappa_j}{\kappa_i}} b_j, \dfrac{\sigma^2/h}{\kappa_i\left(\sum_{j=1}^{n} w_{ij}\right)}\right)$

Table 10: Comparison of the models introduced by Congdon[3], Copras-Burgos and Martinez-Beneito[8] (CB-MB), and the heavy-tailed BYM2 proposal. In our proposal, the unstructured component θ_i is independent of the spatially structured component u_i.

References

[1] Rue H and Held L. *Gaussian Markov random fields: theory and applications*. CRC Press, 2005.

[2] Watanabe S and Opper M. Asymptotic equivalence of Bayes cross validation and widely applicable information criterion in singular learning theory. *Journal of Machine Learning Research* 2010; 11(12): 3571–3594.

[3] Congdon P. Representing spatial dependence and spatial discontinuity in ecological epidemiology: a scale mixture approach. *Stochastic Environmental Research and Risk Assessment* 2017; 31(2): 291–304.

[4] Richardson S, Thomson A, Best N et al. Interpreting posterior relative risk estimates in disease-mapping studies. *Environmental Health Perspectives* 2004; 112(9): 1016–1025.

[5] Stan Development Team. RStan: the R interface to Stan, 2020. URL http://mc-stan.org/. R package version 2.21.2.

[6] Gelman A, Rubin DB et al. Inference from iterative simulation using multiple sequences. *Statistical Science* 1992; 7(4): 457–472.

[7] Vehtari A, Gelman A, Simpson D et al. Rank-normalization, folding, and localization: An improved $\widehat{R}$ for assessing convergence of MCMC. *Bayesian Analysis* 2021; 16(2): 667–718.

[8] Corpas-Burgos F and Martinez-Beneito MA. On the use of adaptive spatial weight matrices from disease mapping multivariate analyses. *Stochastic Environmental Research and Risk Assessment* 2020; 34(3): 531–544.
